# HLSC-Derived Extracellular Vesicles Attenuate Liver Fibrosis and Inflammation in a Murine Model of Non-alcoholic Steatohepatitis

**DOI:** 10.1016/j.ymthe.2019.10.016

**Published:** 2019-10-28

**Authors:** Stefania Bruno, Chiara Pasquino, Maria Beatriz Herrera Sanchez, Marta Tapparo, Federico Figliolini, Cristina Grange, Giulia Chiabotto, Massimo Cedrino, Maria Chiara Deregibus, Ciro Tetta, Giovanni Camussi

**Affiliations:** 1 Department of Medical Sciences, University of Torino, Torino, Italy; 2 Molecular Biotechnology Centre, University of Torino, Torino, Italy; 3 2i3T Società per la Gestione dell’Incubatore di Imprese e per il Trasferimento Tecnologico Scarl, University of Torino, Torino, Italy; 4 Unicyte Srl, Torino, Italy

**Keywords:** human liver stem cells, chronic liver disease, NASH, NAFLD, methionine and choline deprived diet, stem cell-derived EVs

## Abstract

Extracellular vesicles (EVs) are membrane vesicles released virtually by all cell types. Several studies have shown that stem cell-derived EVs may mimic both *in vitro* and *in vivo* the biological effects of the cells. We recently demonstrated that non-alcoholic steatohepatitis (NASH) is inhibited by treatment with human liver stem cells (HLSCs). The aim of the present study was to evaluate whether EVs released by HLSCs influence the progression of NASH, induced by a diet deprived of methionine and choline, in immunocompromised mice. EV treatment was initiated after 2 weeks of diet with a biweekly administration of three different doses. Bio-distribution evaluated by optical imaging showed a preferential accumulation in normal and, in particular, in fibrotic liver. EV treatment significantly improved liver function and reduced signs of liver fibrosis and inflammation at both morphological and molecular levels. In particular, we observed that, out of 29 fibrosis-associated genes upregulated in NASH liver, 28 were significantly downregulated by EV treatment. In conclusion, HLSC-derived EVs display anti-fibrotic and anti-inflammatory effects in a model of chronic liver disease, leading to an improvement of liver function.

## Teaser

Stem cell-derived extracellular vesicles, by modulating pro-fibrotic and pro-inflammatory genes, improve liver function and morphology in an experimental murine model of non-alcoholic steatohepatitis.

## Introduction

Non-alcoholic fatty liver disease (NAFLD) is one of the most common causes of chronic liver disease. NAFLD progression may lead to non-alcoholic steatohepatitis (NASH), characterized by liver inflammation and fibrosis leading to end-stage liver disease, including cirrhosis and hepatocellular carcinoma.^1^^,^^2^ The establishment of anti-fibrotic and anti-inflammatory therapies is one of the major clinical requirement to impinge on liver fibrosis and, especially, NASH.

Human liver stem cells (HLSCs) are an easily obtainable and expandable stem cell population derived from human adult liver cells.^3^^,^^4^ HLSCs share with mesenchymal stromal cells (MSCs) the expression of typical surface markers (CD73, CD29, CD105, CD90, and CD44), the differentiation capacity (osteoblasts), and the immunomodulatory properties.^3^, ^4^, ^5^ Moreover, HLSCs show a specific hepatic commitment as they express typical markers of liver cells such as alpha-feto protein, albumin, and cytokeratins 8 and 18.^3^^,^^4^ HLSCs are negative for the hematopoietic and cytokeratin 19 markers of oval cells and did not express *α*-*Sma*.^4^ HLSCs are able to differentiate *in vitro* into functional hepatocytes and to form islet-like structures.^3^^,^^6^^,^^7^ When tested *in vivo*, HLSCs are able to improve function and morphology in different experimental models of acute liver and renal injuries.^3^^,^^8^^,^^9^ It has been recently demonstrated that HLSCs exhibit anti-fibrotic and anti-inflammatory effects in a murine model of NASH by regulating specific genes without the need for differentiation into mature hepatocytes.^4^

The aim of the present study was to investigate the mechanisms involved in the anti-fibrotic activity of HLSCs. Previous studies have shown that extracellular vesicles (EVs) are involved in the paracrine activity of HLSCs, including anti-fibrotic effects in pre-clinical models of chronic kidney diseases.^10^^,^^11^ Cell-released EVs are a heterogeneous population of membrane vesicles, containing cytosol enclosed into a lipid bilayer. EVs may transfer their cargo (various types of RNAs, proteins, and bioactive lipids) from the producing cell to the recipient cell. EVs derived from different type of stem/progenitor cells may induce regenerative programs in injured organs/tissues by transcriptional or translational modification in injured target cells.^12^^,^^13^

To investigate whether HLSC-released EVs (EV-HLSCs) are implicated in the anti-fibrotic effect of HLSCs, we established a model of NASH induced by a methionine- and choline-deprived diet (MCDD) in immunodeficient mice and started a biweekly treatment with EVs after 2 weeks of diet. Mice were sacrificed at week 4; and functional, morphological, and molecular liver alterations were analyzed.

## Results

### Characterization of EV-HLSCs

**Figure 1:**
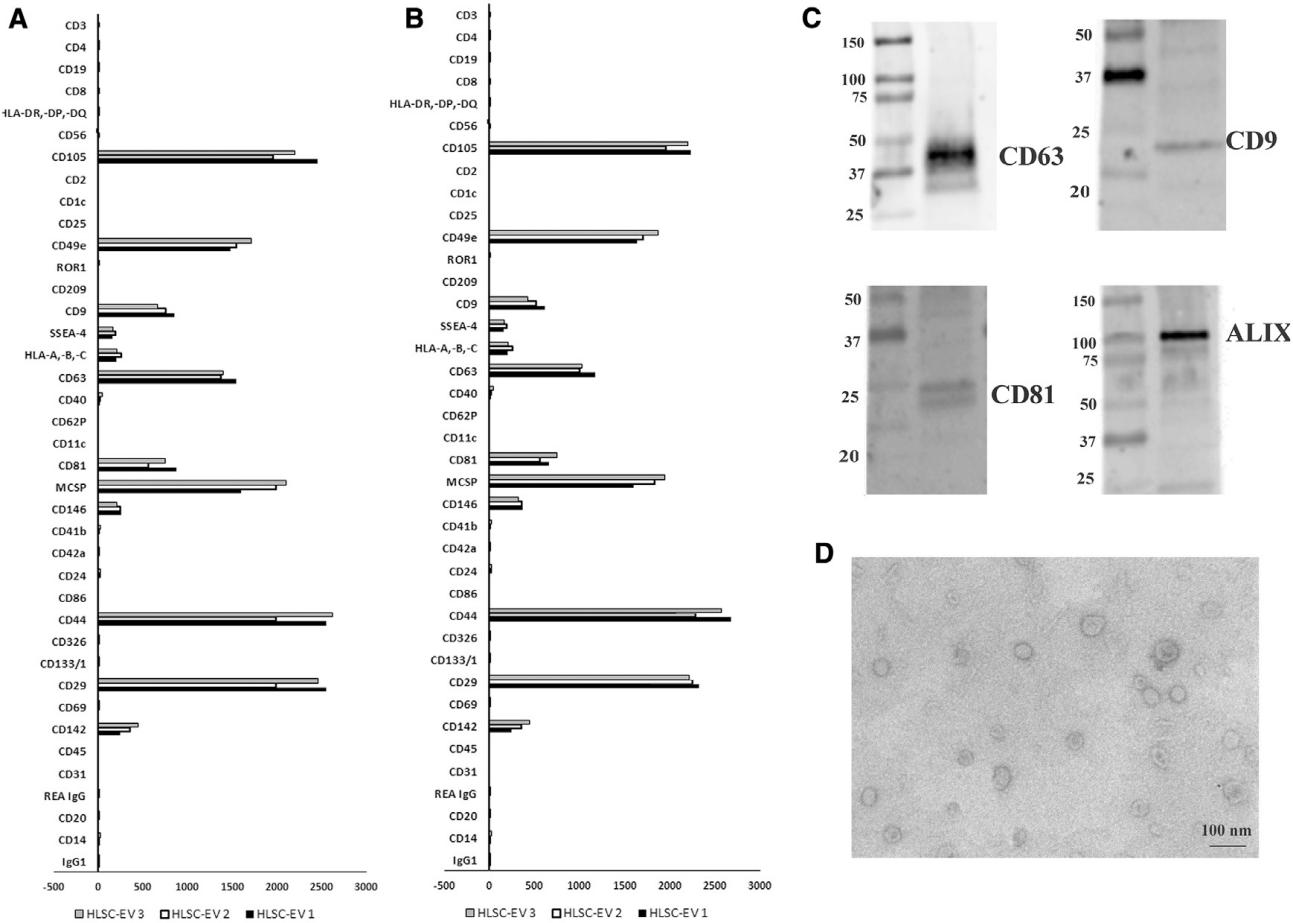
Characterization of EV-HLSCs A multiplex bead-based flow cytometry assay was used to detect EV-HLSC surface signature. 39 multiplexed populations of dye-labeled antibody-coated capture beads were incubated with 3 different EV-HLSC samples. (A and B) Captured EV-HLSCs were counterstained with pan-tetraspanins APC-labeled (A) or with CD29 APC (B) detection antibodies. The graph shows a quantification of the median APC fluorescence values for all bead populations after background correction (medium control values subtracted from measured EV-HLSC values) of three different EV-HLSC preparations. No statistically significant differences were observed among the different EV-HLSC preparations evaluated. (C) Representative western blot analysis of exosomal markers (CD63, CD9, CD81, and Alix) in the EV-HLSCs. (D) Representative micrograph of transmission electron microscopy of EV-HLSCs. EVs were negatively stained with NanoVan (scale bar, 100 nm; magnification, 100,000×).

We evaluated EV expression markers using a recently described multiplex bead-based flow cytometry assay platform for EV characterization.^14^^,^^15^ This assay comprises 39 hard-dried capture bead populations, each of them coated with different monoclonal antibodies against 37 surface antigens and with two isotypic controls. Samples were detected by counterstaining with allophycocyanin (APC)-labeled detection antibodies against specific EV surface markers, such as tetraspanins (CD9, CD63, and CD81). EVs reacted with beads expressing CD9, CD63, and CD81. EVs also expressed high fluorescence intensity for CD29, CD44, CD105, and CD49e. Other markers detected at intermediate to low-positive fluorescence intensity comprised mainly CD142, CD146, SSEA-4, and MCSP (Figure 1). Hematopoietic (CD3, CD4, CD8, CD19, etc.), endothelial (CD31), and epithelial (CD326) markers were not detected in EVs (Figure 1A). When using APC-conjugated anti-CD29 antibody for detection in the same samples, a co-expression of tetraspanin exosomal markers, MSC markers CD29, CD44, CD105, CD49e, CD142, CD146, SSEA-4, and the adhesion molecule MCSP (Figure 1B) was observed. The expression of tetraspanins was confirmed by western blot analysis (Figure 1C).

By transmission electron microscopy, EV preparations showed a homogeneous pattern of nano-sized membrane vesicles (Figure 1D).

### EV-HLSCs Improve Liver Function and Morphology of NASH Mice

**Figure 2:**
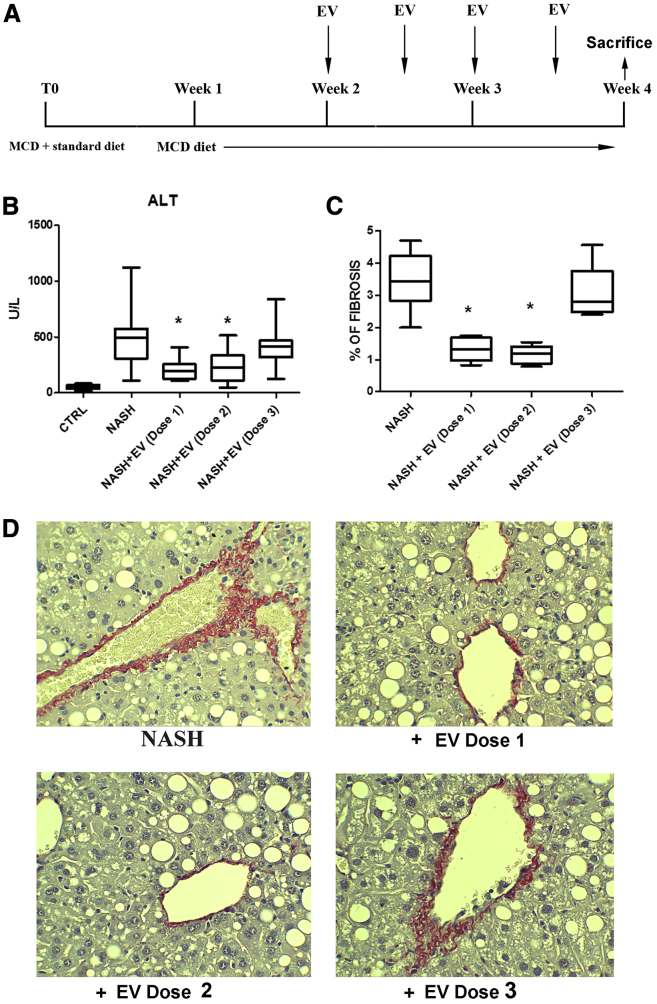
Effect of EV-HLSCs on Liver Function and Morphology (A) Schematic representation of the experimental design to test EV-HLSCs in NASH mice, showing the weeks of feeding with MCCD, of EV-HLSC administration, and of sacrifice. (B) ALT levels expressed as units per liter were measured as a bio-marker of liver cell injury in serum of control mice (CTRL) and of mice fed with MCDD for 4 weeks treated with vehicle alone (NASH) or with iv injection of different doses of EVs (dose 1: 5 × 10^9^ EVs per mouse per injection, n = 9; dose 2: 2.5 × 10^9^ EVs per mouse per injection, n = 9; and dose 3: 2.5 × 10^8^ EVs per mouse per injection, n = 8) and sacrificed at week 4. (C) Histological quantification of fibrosis in MCDD-fed mice injected with different doses of EVs or with vehicle alone (NASH) and sacrificed at week 4 by multiphase image analyses of 10 fields per section (original magnification, 400×). Data shown for ALT and fibrosis represent mean + SD. ANOVA with Newman-Keuls multicomparison test was performed. *p < 0.05, NASH mice injected with doses 1 and 2 of EV-HLSCs versus NASH mice treated with vehicle alone. (D) Representative light microscopy micrographs of liver histology of mice at week 4 of MCDD, treated with injection i.v. of different doses of EV-HLSCs or with vehicle alone (NASH) (original magnification, 400×). Sirius Red staining showed fibrosis in the peri-venous area of NASH mice. Red stain represents collagen fibers considered to be a marker of liver fibrosis.

To evaluate the potential therapeutic ability of EV-HLSCs on liver fibrosis and inflammation, we induced NASH by feeding severe combined immunodeficient (SCID) mice with an MCDD for 4 weeks, as described previously.^4^^,^^16^, ^17^, ^18^, ^19^ Different doses of EVs were administered through intravenous (i.v.) injection twice a week, starting at week 2 of the MCDD (Figure 2A).

Injection of doses 1 and 2 (dose 1: 5 × 10^9^ EVs per mouse per injection; dose 2: 2.5 × 10^9^ EVs per mouse per injection) of EVs induced a significant reduction of alanine aminotransferase (ALT) in the plasma of NASH mice (Figure 2B). Instead, the reduction of aspartate aminotransferase (AST) did not reach the statistical significance (Figure S1A). Serum albumin levels were increased in NASH mice treated with doses 1 and 2 of EVs, compared to NASH mice treated with vehicle alone (Figure S1B). The lower dose tested (dose 3: 2.5 × 10^8^ EVs per mouse per injection) did not improve liver function in NASH-treated mice (Figure 2B; Figure S1). The effect of EVs on liver function, however, was less effective than the one observed with HLSC treatment,^4^ probably because the functional contribution of the liver engrafted HLSCs.

Liver fibrosis, as demonstrated by Sirius Red staining, showed an increase in NASH mice in both peri-venous and peri-portal areas (Figure 2D). Fibrosis was significantly reduced by treatment with doses 1 and 2 but not with dose 3 of EVs (Figure 2C). Treatment with EVs did not attenuate steatosis (Figure S1C).

**Figure 3:**
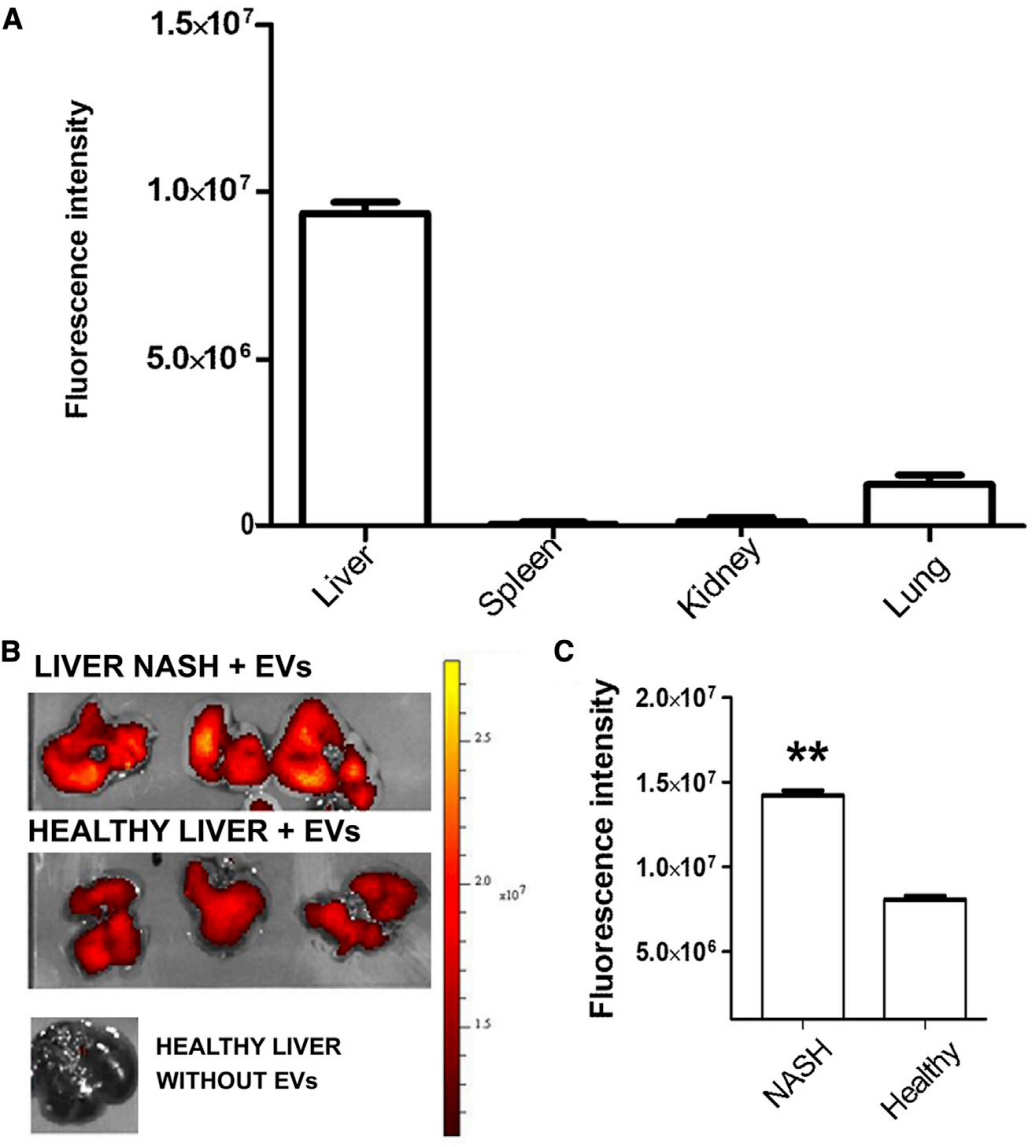
Bio-distribution of EV-HLSCs (A) Quantification of fluorescence intensity in dissected organs of MCDD-fed mice, measured as average radiance ± SD at 3 h post-EV-HLSC administration (n = 6). (B) Representative images obtained by optical imaging of livers of NASH and healthy mice, collected 3 h after EV-HLSC or vehicle administration. (C) Quantification of fluorescence intensity in livers of NASH and healthy mice injected with fluorescent EV-HLSCs. **p < 0.01, NASH mice versus healthy control mice, Student’s t test.

Bio-distribution of EVs was performed by i.v. injection of labeled EVs in healthy and NASH mice. The organ uptake was assessed 3 h after administration. Accumulation of EVs was most prominent in the liver and at a low level in lungs, whereas no signal was observed in spleen and kidney (Figure 3A). Comparing the localization of fluorescent EVs in liver of NASH versus control healthy mice, we observed a significant accumulation of fluorescent EVs in the fibrotic liver (Figures 3B and 3C). Moreover, in comparing the fluorescent signaling in lungs of NASH versus control healthy mice, a significant lower accumulation of fluorescent EVs was observed in NASH mice (Figure S2). This could be due to an enhanced liver uptake of EVs in NASH mice.

### EV-HLSC Treatment Downregulates Pro-fibrotic and Pro-inflammatory Genes in the Liver of NASH Mice

**Figure 4:**
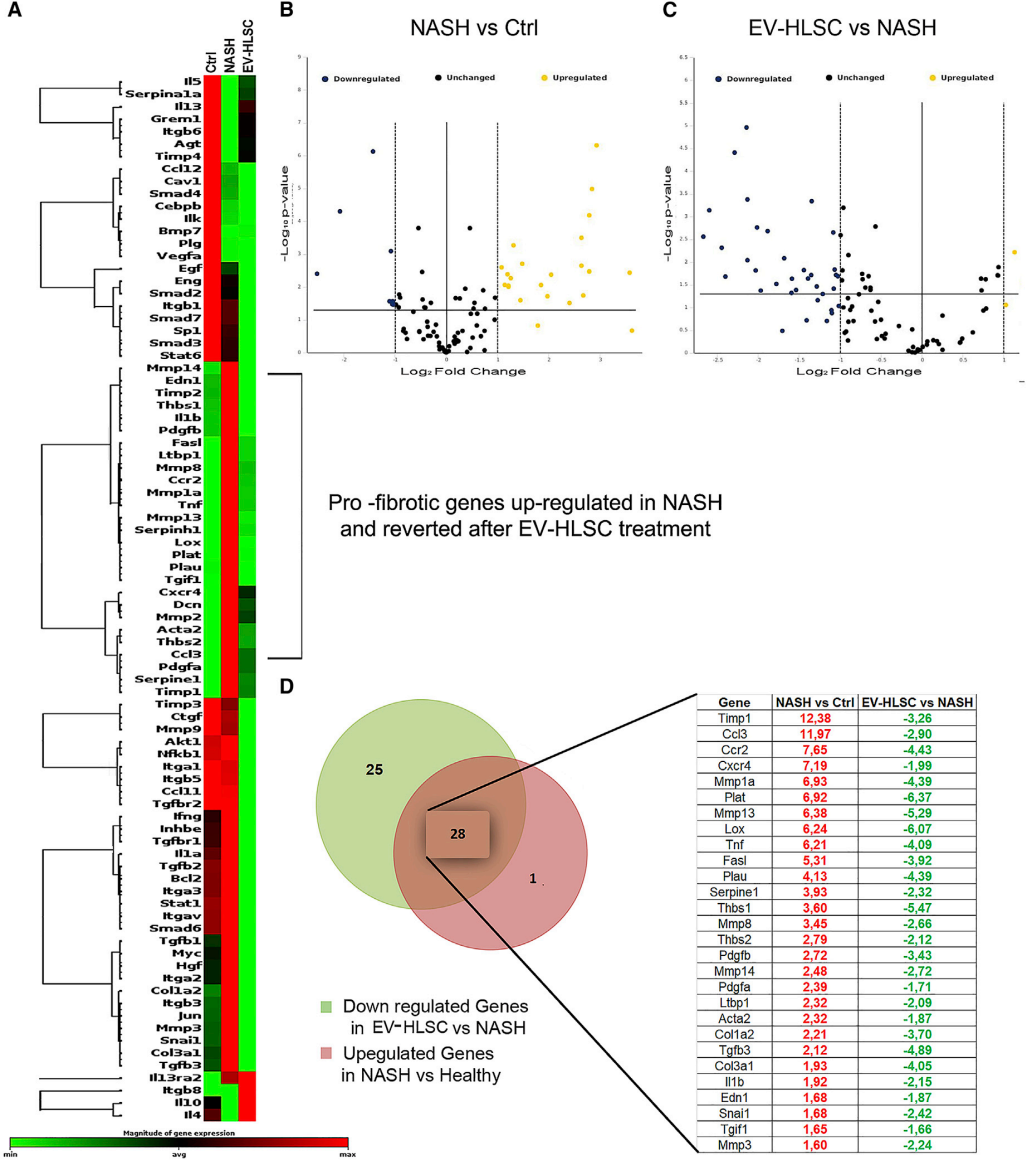
Expression of Fibrosis-Related Genes in NASH Mice Treated or Not with EV-HLSCs Analyzed through a Fibrosis RT2 Profiler PCR Array (QIAGEN) (A) Heatmap displaying hierarchical clustering of the entire dataset of expressed genes and indicating co-regulated genes across three different experimental groups: healthy (CTRL) mice, NASH mice, and EV-HLSC-treated mice. Clustering analysis showed that genes upregulated in NASH mice belong to pro-fibrotic and pro-inflammatory pathways; EV-HLSC treatment was able to restore their expression to healthy levels (n = 4 mice analyzed for each experimental condition). (B and C) The volcano plots identify significant changes in gene expression between groups: increased number of upregulated genes in NASH mice compared to healthy mice (B) and evident downregulation of gene expression in EV-HLSC-treated mice compared to the NASH group (C). The volcano plot displays the statistical significance (p < 0.005) versus fold change on the y and x axes, respectively. (D) Venn diagram and list of pro-fibrotic and pro-inflammatory genes regulated in NASH mice treated or not treated with HLSC EVs. The expression levels of genes are presented as fold regulation values (those greater than 1.6 are indicated in red, and those less than −1.6 are indicated in green) for NASH mice compared to those for controls (NASH vs Ctrl column) and for treated mice compared to those for the NASH group (EV-HLSC vs NASH column). Complete data are provided in Table S1.

We investigated the possibility that the improvement in liver function and morphology after EV treatments may depend on the regulation of genes that are associated with the induction of fibrosis. To this purpose, liver tissue obtained from NASH mice, treated or not treated with dose 2 of EVs, was subjected to RNA isolation, and the Mouse Fibrosis RT^2^ Profiler PCR Array was performed. The gene expression profiles of the healthy mice and NASH mice treated or not treated with EVs were compared. Figure 4A shows the heatmap of genes expressed in the experimental groups. Clustering analyses revealed significant changes in the expression of genes in NASH mice. Twenty-nine mRNAs out of 84 key genes were upregulated and 9 were downregulated in NASH mice compared to those from the healthy group (Figure 4B; Table S1). The majority of these genes encoded extracellular matrix (ECM) remodeling enzymes, transforming growth factor beta 1 (*Tgf-β1*) signaling molecules, and inflammatory cytokines involved in the development of liver fibrosis (Figure 4A). Comparing to NASH mice, 53 genes were downregulated by EV-HLSC treatment (Figure 4C; Table S1). Interestingly, 28 out of 29 mRNAs upregulated in NASH mice were significantly reverted by EV treatment (Figure 4D). The list of reverted genes included alpha smooth muscle actin (*α-Sma*), Collagen 1a1 (*Col1α1*), *Tgf-β1*, and the gene latent-transforming growth factor beta-binding protein 1 (*Ltbp1*). All these genes are known to be involved in fibrosis. EV treatment also modulated genes involved in tissue remodeling and in inflammation, such as TIMP metallopeptidase inhibitor 1 (*Timp-1*), matrix metallopeptidases (*Mmp-1a*, *-13*, *-14*, and *-8*), tumor necrosis factor alpha (*Tnf*), and interleukin-1 beta (*IL-1β*) (Figure 4D).

**Figure 5:**
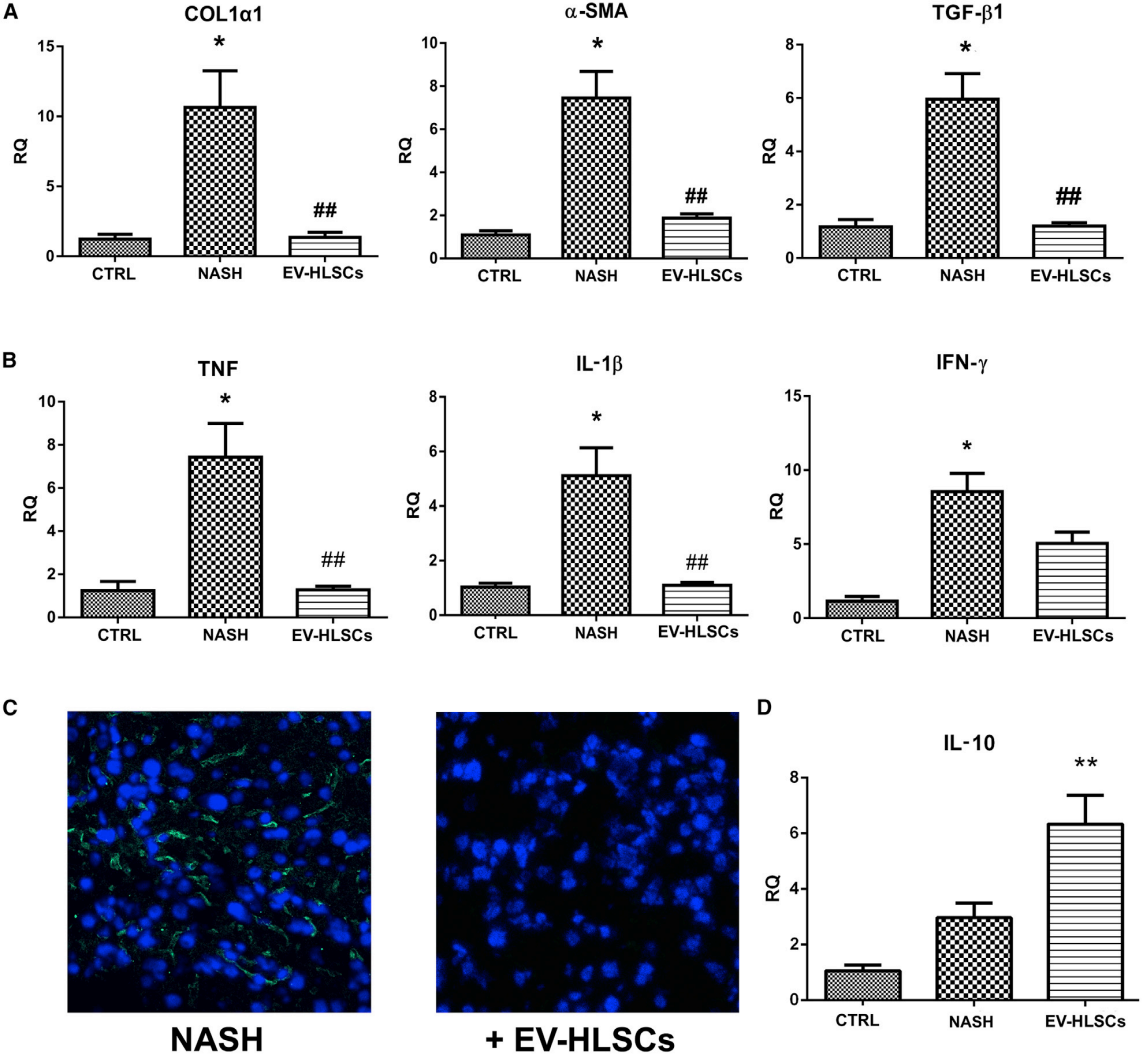
Effect of EV-HLSCs on Liver Fibrosis and Inflammation (A) Gene expression levels of fibrotic markers (*Col1α1*, *α-Sma*, and *Tgf-β*1) in livers of healthy mice (CTRL) and of NASH mice injected with dose 2 of EV-HLSCs (EV-HLSC) or with vehicle alone (NASH). *p < 0.05, NASH mice treated with vehicle (NASH) versus normal healthy mice (CTRL); ^##^p > 0.01, NASH mice treated with vesicles (EV-HLSCs) versus NASH mice injected with vehicle alone (NASH). (B) Gene expression levels of pro-inflammatory cytokines (*Tnf, IL-1β* and *Ifn-γ*) in livers of healthy mice (CTRL) and of NASH mice treated with dose 2 of EV-HLSCs (EV-HLSCs) or with vehicle alone (NASH). For fibrotic and pro-inflammatory genes, data are expressed as relative quantification using the ΔΔCt method. Normalization was made using GAPDH as a housekeeping gene. An ANOVA with Newman-Keuls multicomparison test was performed. (C) Representative micrographs of liver cryosections from NASH mice injected or not injected with dose 2 of EV-HLSCs stained for CD45 to identify the presence of inflammatory cells. Infiltrates of inflammatory CD45-positive cells (green) were present only in NASH mice that did not receive EV administration. Nuclei were counterstained with DAPI (blue). Original magnification, 400×. (D) Gene expression levels of anti-inflammatory cytokine *IL-10* in livers of healthy mice (CTRL) and of NASH mice treated with dose 2 of EV-HLSCs (EV-HLSCs) or with vehicle alone (NASH). Data are expressed as relative quantification using the ΔΔCt method. Normalization was made using GAPDH as a housekeeping gene. An ANOVA with Newman-Keuls multicomparison test was performed. **p < 0.01, NASH mice treated with vesicles (EV-HLSCs) versus NASH mice injected with vehicle alone (NASH).

Real-time PCR confirmed that NASH mice had significantly upregulated levels of the pro-fibrotic genes *α-Sma*, *Col1a1* and *Tgf-β1* (Figure 5A) and of the pro-inflammatory genes *Tnf*, *IL-1β*, and interferon gamma (*Ifn-γ*) (Figure 5B). NASH mice treated with EVs had a significant reduction in the expression levels of all fibrotic and pro-inflammatory genes tested (Figures 5A and 5B). The anti-inflammatory effect of EV treatment was also indicated by the reduction of inflammatory cells accumulated in the liver, as seen by immunofluorescence staining with the leucocyte marker CD45. Whereas inflammatory infiltrates were present in the liver of NASH mice, almost no leukocytes were detected in mice treated with EVs (Figure 5C). The increase of interleukin 10 (*IL-10*) expression level in NASH mice treated with EVs confirmed the anti-inflammatory effect of EVs (Figure 5D).

### EV-HLSC Protein Cargo May Modulate Inflammation

**Figure 6:**
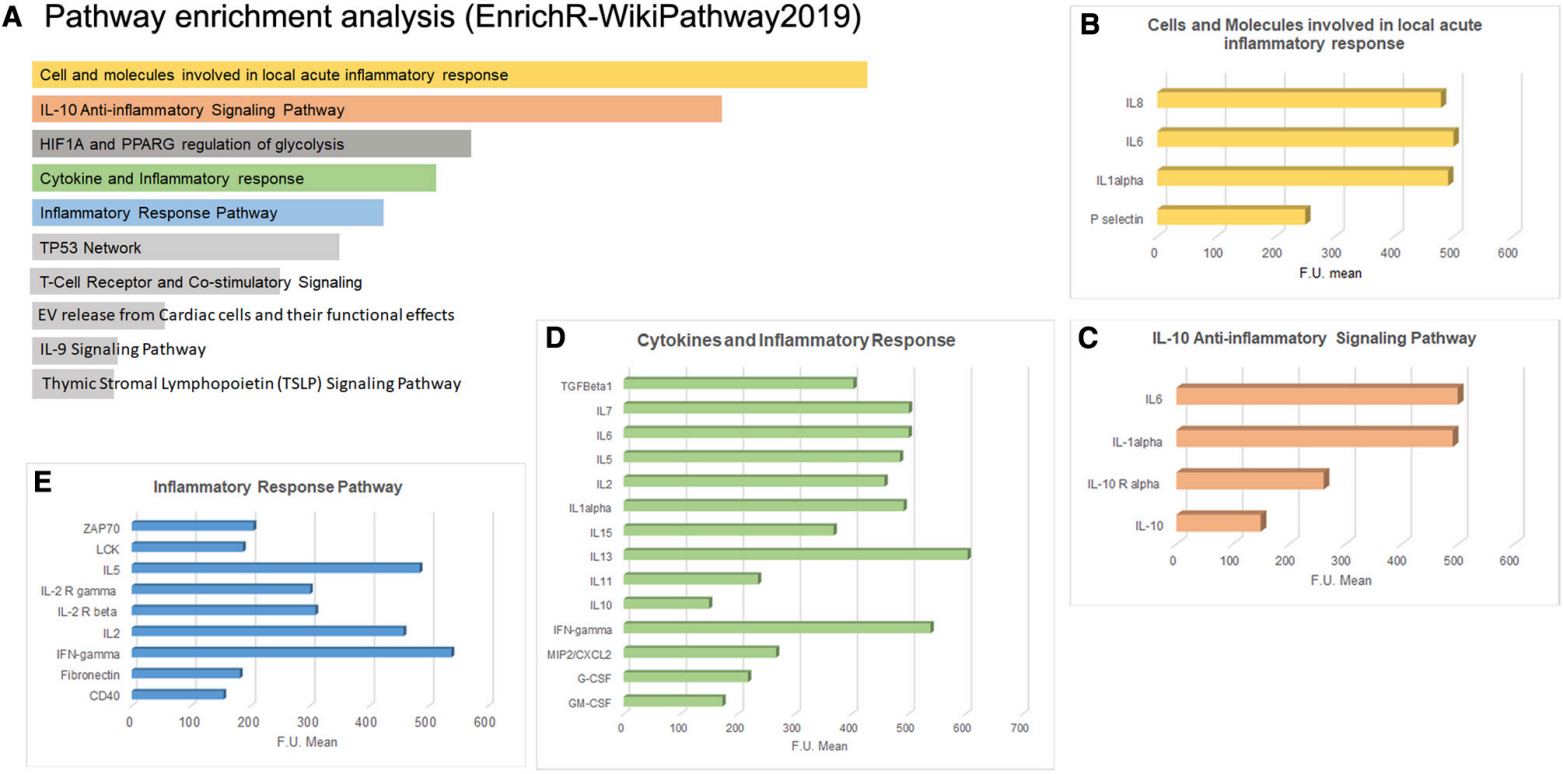
Pathway Enrichment Analysis of Proteins Vehicled by EV-HLSCs (A) Representative bar graph showing the main pathways in which the 251 proteins vehicled by EV-HLSCs, identified by an antibody-based protein array, are involved. Most of them are related to inflammation and cytokine pathways. Data are ranked according to *Z* score and are reported in Table S2. (B–E) Bar graph showing the fluorescence intensity mean (F.U. mean) of the different proteins clustered in the main enriched pathways involved in inflammation: (B) proteins of local acute inflammatory response; (C) IL-10 anti-inflammatory signaling pathway; (D) cytokines and inflammatory response; and (E) inflammatory response pathway. The complete list of proteins with FU mean quantitation and pathway enrichment analysis are available in Table S3.

Protein analysis showed the presence of 251 proteins in EVs (Table S2). Most of the molecules identified are cytokines and growth factors. Moreover, among the enriched EV proteins, we observed the presence of transmembrane tyrosine kinase receptors, MMPs, some enzymes (glutatione reductase, creatine kinase, and aspartate beta-hydroxylase), and transcription factors. Pathway enrichment analysis showed that the most enriched pathways were related to cytokine and inflammatory response, IL-10 anti-inflammatory activity, phosphatidylinositol 3-kinase (PI3K) pathways, and the p53 pathway (Figure 6; Table S3).

## Discussion

Results of the present study show that EV-HLSCs exerted beneficial effects on liver morphology, ameliorating fibrosis and inflammation by reprogramming liver gene expression. Previous studies have shown that EVs may induce epigenetic changes into recipient cells by transferring different molecules from the originator cells. It has been therefore suggested that stem cell-derived EVs may be exploited to promote tissue regeneration. Previous studies demonstrated that EVs obtained by MSCs of different origin improve acute liver injuries and increase survival in different lethal models of liver damages.^20^^,^^21^ Moreover, MSC-derived EVs were shown to ameliorate hepatic ischemia reperfusion injury by modulating the inflammatory response^22^^,^^23^ and by improving hepatic oxidant injury in toxic^24^^,^^25^ and ischemic^26^ models. EVs derived from different stem cells were also studied in different pre-clinical models of chronic liver injuries. MSC-derived EVs were able to reduce fibrosis in CCl4- and thioacetamide-induced chronic liver injures by alleviating hepatic inflammation and collagen deposition and by inhibiting epithelial-to-mesenchymal transition.^27^, ^28^, ^29^, ^30^ Moreover, EVs derived from serum reduced liver fibrosis in mice treated with CCl4 or thioacetic acid by improving liver function, reducing hepatocyte apoptosis, and suppressing inflammation.^31^ These recent papers indicate that EVs can be exploited for therapy in liver diseases. EVs show a superior safety profile to cell-based therapy, as they pass biological barriers and act as effective transporters of different molecules.^32^ Interestingly, EVs derived from HLSCs preferentially localized in liver—in particular, in fibrotic areas after systemic administration, as seen by optical imaging (OI). Different amounts of EV-HLSCs have been tested in NASH mice. We found that 2.5 × 10^9^ EVs, injected twice a week starting at week 2 of an MCDD, are sufficient to obtain amelioration of liver function and morphology and a significant reduction of the percentage of fibrosis. At the molecular level, we found that 29 genes related to fibrosis and inflammation were upregulated in the liver of NASH mice, including transcripts for ECM remodeling enzymes (*mmp*-*1a*, -*3*, -*8*, *-14*, and *Timp-1*), *Tgf-β* and *Tgf-β* signaling molecules, inflammatory cytokines (*IL-1β* and *Tnf-α*) and chemokines (*Ccl3* and *Ccl2*), and chemokine receptor (*CXCR4*). In the liver of NASH mice treated with EVs, 28 out of 29 pro-fibrotic genes were downregulated. Notably, the mRNA expression level of lysyl oxidase (*Lox*), which is considered a serum biomarker of liver fibrosis in patients with NAFLD,^33^ reverted in mice treated with EVs, along with the expression levels of *Col1α1*, *Tgf-β1*, *Ltbp-1*, and *α-Sma*. In particular, *Ltbp-1*, which encodes for the protein responsible for activating *Tgf-β1* from its latent form to the active form and which is considered fundamental for fibrosis progression, was downregulated by EV treatment.^34^

A key factor in the progression of NASH is the inflammation.^16^, ^17^, ^18^, ^19^ An infiltration of CD45-positive cells was observed in mice maintained with an MCDD for 4 weeks. NASH mice treated with EV-HLSCs had a significant reduction of CD45 cell accumulation within the liver, indicating the anti-inflammatory and immunomodulatory effects of EVs as detected at the molecular level. Moreover, the expression of IL-10, an anti-inflammatory and immunomodulatory cytokine,^35^ was significantly enhanced after EV treatment.

We previously showed in two different models of chronic kidney diseases that the anti-fibrotic effect of EV-HLSCs is linked to the presence of microRNA (miRNA) patterns targeting the fibrotic genes.^10^^,^^11^ In particular, we found that EV-HLSCs contain miRNA-29a, the let-7 family, miRNA-30a, miRNA-24, and miRNA-21, which are known to target Collagen I, Snail, and the FAS ligand.^11^

In the present study, we performed proteomic analyses of EV-HLSCs showing the presence of several anti-inflammatory proteins that may contribute to the final anti-fibrotic effect by downregulating inflammation. Therefore, EV-associated proteins may implement the biological activity of regulatory RNAs present in the EVs.^10^^,^^11^

In conclusion, the results of the present study indicated that EVs released from HLSCs exert anti-inflammatory and anti-fibrotic effects in a model of chronic liver disease by carrying molecules that may modulate genes involved in fibrosis. Fibrosis is an abnormal mechanism of tissue repair after repeated and sustained injuries that has common pathways in different organs. Therefore, taking into account the properties of EV-HLSCs, we can speculate that this may represent a potential therapeutic approach aimed to modify the abnormal response of different tissues to injury.

## Materials and Methods

### HLSC Culture

HLSCs were obtained and cultured as previously described.^3^^,^^4^ Briefly, HLSCs were generated by Anemocyte International (Gerenzano, Italy) from a 10- to15-mm liver fragment obtained from a liver donor, according to the standard criteria of Centro Nazionale Trapianti, as described previously.^3^^,^^4^ The liver fragment was enzymatically digested with good manufacturing practice (GMP)-grade collagenase (NB 1, 0.6 mg/mL) and neutral protease (NBI, 0.73 mg/mL) for 30 min at 37°C. The liver cell suspension was washed (400 × g for 10 min) and cultured (2.5 × 10^5^/mL in a T75 flask with 10 mL per flask) in the presence of minimal essential medium (α-MEM; Lonza, Basel, Switzerland) supplemented with 10% fetal calf serum (GIBCO, Cambrex), 10 ng/mL human recombinant epidermal growth factor (Miltenyi, Bergisch Gladbach, Germany), 10 ng/mL human recombinant basic fibroblast growth factor (Miltenyi, Bergisch Gladbach, Germany), 2 nM L-glutamine (Lonza), and 100 U/mL penicillin/streptomycin (Sigma, St. Louis, MO, USA) and maintained in a humidified 5% CO_2_ incubator at 37°C. After 2 weeks of culture, cells were seeded at a density of 2.5 × 10^5^ cells per flask (T75) in the same culture medium for expansion.

### EV-HLSCs: Purification and Characterization

EVs were obtained from supernatants of sub-confluent HLSCs cultured in serum-free α-MEM (EuroClone, Pero, Italy) for 18 h. Viability of cells at the time of supernatant collection was 98%, as confirmed by trypan blue exclusion. After the removal of cell debris and apoptotic bodies by centrifugation at 3,000 × *g* for 20 min and by microfiltration with 0.22-μm filters, EVs were purified by ultracentrifugation at 100,000 × *g* for 2 h at 4°C (Optima L-90 K; Beckman Coulter, Fullerton, CA, USA). The pellet of EVs obtained was resuspended in RPMI supplemented with 1% DMSO and stored at −80°C until use for subsequent studies.

For bio-distribution experiments, EV-HLSCs were labeled during ultracentrifugation with DiD fluorescent dye (Thermo Fisher Scientific, Waltham, MA, USA) as previously described.^36^ One micromolar of Vybrant Cell Tracers DiD was added during the ultracentrifugation procedure. Then, labeled EVs were washed twice by ultracentrifugation in PBS (Lonza).

EV-HLSC number was determined using the NanoSight NS300 system (NanoSight, Amesbury, UK). EV-HLSC preparations were diluted (1:200) in sterile saline solution and analyzed using the NanoParticle Tracking Analysis (NTA) System with the NTA 3.2 Analytical Software as described previously.^36^

EV-HLSCs were characterized by cytofluorimetric analysis. Different EV-HLSC preparations (n = 3) were subjected to bead-based multiplex analysis by flow cytometry (MACSPlex Exosome Kit, human, Miltenyi Biotec).^14^^,^^15^ Approximately 1 × 10^9^ EV-HLSCs, quantified by NTA, were diluted with MACSPlex buffer (MPB) to a final volume of 120 μL and loaded into a 1.5-mL tube. After this, 15 μL MACSPlex Exosome Capture Beads (containing 39 different antibody-coated bead subsets) was added to each tube. For counterstaining of EVs bound by capture beads with detection antibodies, 5 μL each of APC-conjugated anti-CD9, anti-CD63, and anti-CD81 detection antibodies was added to each tube and then incubated in an orbital shaker for 1 h at 450 rpm at room temperature, protected from light. In this study, we mostly used a mixture of all three antibodies (pan-tetraspanin) in order to cover most EVs being present in the samples. In selected experiments, we used 5 μL APC-conjugated anti-CD29 (Miltenyi Biotec) antibody instead of the anti-tetraspanin antibodies. To wash the beads, 1 mL MPB was added to each tube and washed at 3,000 × g for 5 min. This was followed by another washing step with 1 mL MPB; incubation in an orbital shaker at 450 rpm, protected from light for 15 min at room temperature; and then washing at 3,000 × g for 5 min. After washing, 1 mL of the supernatant was carefully aspirated, leaving about 150 μL in the tubes, ready to be acquired.

Flow-cytometric analysis was performed with a CytoFLEX flow cytometer (Beckman Coulter, Brea, CA, USA). Approximately 5,000–8,000 single-bead events have been recorded per sample. Median fluorescence intensity (MFI) for all 39 capture bead subsets were background corrected by subtracting respective MFI values from matched media controls that were treated exactly like EV-containing samples (buffer/medium + capture beads + antibodies). All bead populations can be identified and gated based on their respective fluorescence intensity according to the manufacturer’s instructions.

Transmission electron microscopy was performed on EVs placed on 200-mesh nickel formvar carbon-coated grids (Electron Microscopy Science, Hatfield, PA, USA) and left to adhere for 20 min, as previously described.^37^ The grids were then incubated with 2.5% glutaraldehyde containing 2% sucrose and, after washings in distilled water, the EVs were negatively stained with NanoVan (Nanoprobes, Yaphank, NY, USA) and observed using a Jeol JEM 1010 electron microscope (Jeol, Tokyo, Japan).

### Western Blot Analysis

Protein samples at a concentration of 10–30 μg were separated in 8% or 4%–15% gradient SDS-PAGE gels under reducing conditions and electroblotted onto 0.2-mm nitrocellulose membranes (GE Healthcare Life Sciences, Marlborough, MA, USA). The membranes were blocked in Tris-buffered saline-Tween 20 (TBS-T; 25 mM Tris [pH 8.0], 150 mM NaCl, and 0.05% Tween-20) containing 5% (w/v) non-fat dried milk for 1 h. After blocking, membranes were probed overnight with mouse anti-CD63, mouse anti-Alix (both from Santa Cruz Biotechnology, Santa Cruz, CA, USA), rabbit anti-CD9 (Abcam), and mouse anti-CD81 (Becton Dickinson). After extensive washings with TBS-T, the blots were incubated with the appropriate peroxidase-conjugated secondary antibodies for 1 h at room temperature. Following incubation, the membranes were washed extensively with TBS-T, probed with enhanced SuperSignal West Femto Maximum Sensitivity Substrate (Thermo Fisher Scientific), and detected with the ChemiDoc system (Bio-Rad, Hercules, CA, USA).

### *In Vivo* Murine Model

Animal studies were conducted in accordance with the NIH Guide for the Care and Use of Laboratory Animals. All procedures were approved by the Italian Health Ministry (authorization number: 419/2016-PR).

To evaluate the ability of EV-HLSCs to improve liver fibrosis and inflammation, we induced NASH by continuously feeding the mice with an MCDD, as previously reported.^4^^,^^16^, ^17^, ^18^, ^19^ Male SCID mice (Charles River Laboratories, Wilmington, MA, USA), 10 weeks old, became accustomed to an MCDD (MP Biomedicals, Eschwege, Germany) through a mixture of standard and MCDD chow for 1 week. Thereafter, the full MCDD was given. Different amounts of EV-HLSCs were administered via i.v. injection (tail vein) twice a week, starting at week 2 of the diet (Figure 2), when fibrosis and inflammation have been established in the liver. Each mouse received a total of four EV injections. Different doses of EVs were tested: dose 1 (n = 9 mice), 5 × 10^9^ EVs per mouse per injection; dose 2 (n = 9 mice), 2.5 × 10^9^ EVs per mouse per injection; and dose 3 (n = 8 mice), 2.5 × 10^8^ EVs per mouse per injection. The NASH control group was injected with vehicle alone (PBS; n = 23 mice). Control animals (n = 8) were fed with standard diet. All animals were sacrificed at week 4, and blood and liver were recovered for biochemical, histological, and molecular analyses.

### *In Vivo* Bio-distribution of EV-HLSCs

Mice fed for 3 weeks with an MCDD and healthy mice were administered an i.v. injection of 3 × 10^10^ fluorescent EV-HLSCs, and their localization was monitored by OI (n = 6 per group). All the images have been acquired with the IVIS 200 small animal imaging system (PerkinElmer, Waltham, MA, USA) using an excitation filter at 640 nm and an emission filter at 700 nm.

Fluorescence analysis was performed by OI on dissected organs 3 h post-EV injection. Fluorescence emission was normalized to photons per second per centimeter squared per steradian (p/sec/cm^2^/sr) as previously described.^36^ The fluorescence signal in livers was quantified in region of interest (ROI) drawn freehand. Data were expressed as the average radiance ± SD. Images were acquired and analyzed using Living Image 4.0 software (PerkinElmer).

### Histological Analyses

Liver morphology was evaluated through formalin-fixed paraffin-embedded tissue staining. Paraffin kidney sections (5 μm thick) were routinely stained for microscopic evaluation with H&E (Merck) or Sirius Red for collagen detection.

Liver fibrosis was quantified by measuring collagenous fibrotic areas stained in red (sections stained with Sirius Red) in 10 random fields per section from images taken at a magnification of 400×, using multiphase image analyses with ImageJ software v1.49.^38^ The surface area occupied by steatosis vacuoles was quantified in 10 random fields per section from images taken at a magnification of 200×, using multiphase image analyses with ImageJ software. Immunofluorescence was performed on 5-μm-thick cryostat sections. Sections were stained with mouse anti-CD45 (Biorbyt, San Francisco, CA, USA) antibody for 2 h at 4°C. Rabbit anti-mouse Alexa Fluor 488 (Molecular Probes) was used as secondary antibody. Hoechst 33258 dye (Sigma) was added for nuclear staining. Confocal microscopy analysis was performed using a Zeiss LSM 5 Pascal model confocal microscope (Carl Zeiss International).

### Molecular Analyses

Total RNA was extracted from liver tissue of control or NASH mice treated with or without EV-HLSCs using TRIzol Reagent (Ambion, Thermo Fisher Scientific, Waltham, MA, USA), according to the manufacturer’s instructions. The TRIzol solutions were homogenized in a Bullet Blender instrument (Next Advance, New York, NY, USA) at a speed of 8 rpm for 3 min using 0.5-mm zirconium oxide beads and centrifuged at 12,000 × g for 15 min at 4°C. Supernatant from homogenized tissue was used to isolate RNA that was quantified spectrophotometrically (Nanodrop ND-2000, Thermo Fisher Scientific).

A total of 12 samples, four mice per experimental group, were retro-transcribed with the RT^2^ First Strand Kit, and gene expression was analyzed using the Mouse Fibrosis RT^2^ Profiler PCR Array (PAMM-120ZC; QIAGEN, Hilden, Germany) following the manufacturer’s protocol. The analysis was performed with the online QIAGEN software using global normalization. Fold regulation expressions with respect to the control or NASH group were calculated for all samples using the ΔΔCt method.

Moreover, to confirm the expression of specific gene expression, quantitative real-time PCR was performed as described previously.^4^ Briefly, 5 or 10 ng cDNA, retrotranscribed from RNA using the High Capacity cDNA Reverse Transcription Kit (Applied Biosystems, Foster City, CA, USA), were used in 20 μL reaction mixture containing sequence-specific oligonucleotide primers (purchased from MWG-Biotech, Eurofins Scientific, Brussels, Belgium) and the Power SYBR Green PCR Master Mix (Applied Biosystems) and were analyzed with StepOne Plus Real-Time PCR System (Applied Biosystems). GAPDH was used as the housekeeping gene to normalize RNA inputs. Fold change expressions with respect to control were calculated for all samples using the ΔΔCt method. The primers used for quantitative real-time PCR are reported in Table 1.

**Table 1:** Real Time PCR Specific Primers and Characteristics of Amplicons

Gene	Sense (5′ → 3′)	Antisense (5′ → 3′)
*m-Col1α1*	ACCTTGTTTGCCAGGTTCAC	ATCTCCCTGGTGCTGATGGAC
*m-Tgf-β*1	GCAACAATTCCTGGCGTTACC	CGAAAGCCCTGTATTCCGTCT
*m-α-Sma*	CATCTCCGAAGTCCAGCACA	GACGCACCACTGAACCCTAA
*m***-***IL-1β*	CAACCAACAAGTGATATTCTCCATG	GATCCACACTCTCCAGCTGCA
*m-Ifn-γ*	GAGCCAGATTATCTCTTTCATCC	GTTGTTGACCTCAAACTTGG
*m-Tnf*	CATCTTCTCAAAATTCGAGTGACAA	TGGGAGTAGACAAGGTACAACCC
*m-IL-10*	GACTTTAAGGGTTACTTGGGTTGC	TCCTGAGGGTCTTCAGCTTCTC
*m-GADPH*	TGTCAAGCTCATTTCCTGGTATGA	TACTCCTTGGAGGCCATGT

### EV-HLSC Protein Content Analysis

EV-HLSC protein content was analyzed with the Human L1000 Array kit (RayBiotech, Peachtree Corners, GA, USA). EV-HLSCs from two different pools were ultracentrifuged for 2 h at 100,000 × *g* at 4°C and then lysed with the lysis buffer provided by the kit following the manufacturer’s instructions. Protein quantification was obtained with the Pierce BCA Protein Assay Kit (Thermo Fisher Scientific). Approximately 25 μg total protein was hybridized onto a glass slide according to the manufacturer’s protocol. Fluorescence intensity was acquired, and background signal was subtracted for each point. Cutoff threshold was set at 150 fluorescent units (FUs). Data are expressed as FUs ± SD.

### Pathway Enrichment Analysis

A list of protein vehicles by EV-HLSCs was subjected to bioinformatics analysis by EnrichR^39^^,^^40^ online software. The software provided different databases, and WikiPathways 2019 was chosen because it was the most updated one. Pathways were listed and ranked after Fisher’s exact test was performed for many random gene sets in order to compute a mean rank and SD from the expected rank for each term in the gene-set library and finally calculating a *Z* score to assess the deviation from the expected rank.

### Statistical Analyses

Data analyses were performed using GraphPad Prism 6.0. Results are expressed as mean ± SD. Statistical analyses were performed by using an ANOVA with a Newman-Keuls test and Student’s t test for bio-distribution experiments. A p value of < 0.05 was considered significant.

## Author Contributions

S.B., M.B.H.S., and M.C. performed *in vivo* studies. S.B. performed cytofluorimetric characterization of EVs. C.P. and G. Chiabotto performed molecular tissue analysis. S.B. and M.B.H.S. performed histological analyses. M.T. and F.F. performed proteomic analyses of EVs. M.T. performed bioinformatics analyses. C.G. performed optical imaging experiments. M.C.D. performed western blot and electron microscopy analyses of the EV preparations. C.T. performed data interpretation and revised the manuscript. S.B. and G. Camussi performed study design, data interpretation, and manuscript writing. All authors approved the submitted the manuscript.

## Conflicts of Interest

C.T. is a full-time employee of Unicyte (Torino, Italy) and contributed to the study as a researcher. G. Camussi is a component of the scientific advisory board of Unicyte. M.B.H.S. and G. Camussi are named inventors in a related patent (WO2006126219-A1). The other authors declare no competing interests.

## Supplemental Information

Document S1. Figures S1–S3 and Tables S1 and S2

Table S3. Pathway Enrichment Analyses of Proteins Carried by EV-HLSCs

Document S2. Article plus Supplemental Information
