# Supplementary material for: HLSC-Derived Extracellular Vesicles Attenuate Liver Fibrosis and Inflammation in a Murine Model of Non-alcoholic Steatohepatitis
Source: Mol Ther. 2019 Oct 28;28(2):479–89. doi: 10.1016/j.ymthe.2019.10.016 (PMC7001005; doi:10.1016/j.ymthe.2019.10.016)
Supplement: Document S1. Figures S1–S3 and Tables S1 and S2 [file mmc1.pdf]

YMTHE, Volume *28*

Supplemental Information

HLSC-Derived Extracellular Vesicles Attenuate Liver Fibrosis and Inflammation in a Murine Model of Non-alcoholic Steatohepatitis

Stefania Bruno, Chiara Pasquino, Maria Beatriz Herrera Sanchez, Marta Tapparo, Federico Figliolini, Cristina Grange, Giulia Chiabotto, Massimo Cedrino, Maria Chiara Deregibus, Ciro Tetta, and Giovanni Camussi

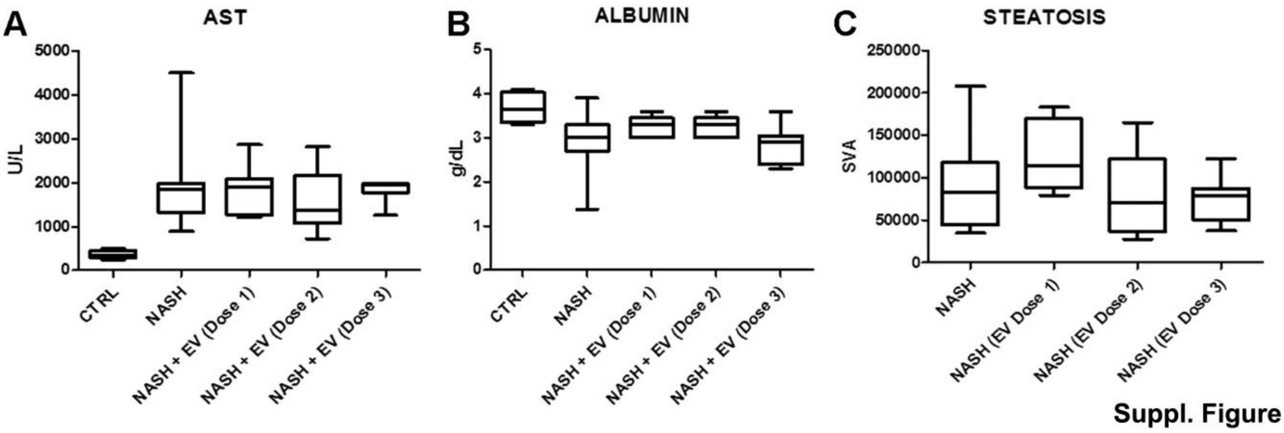

Suppl. Figure 1

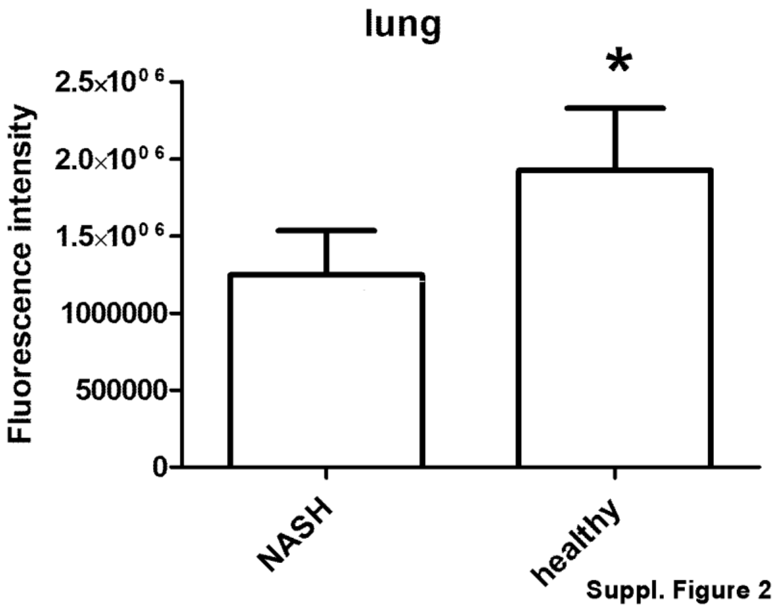

Suppl. Figure 2

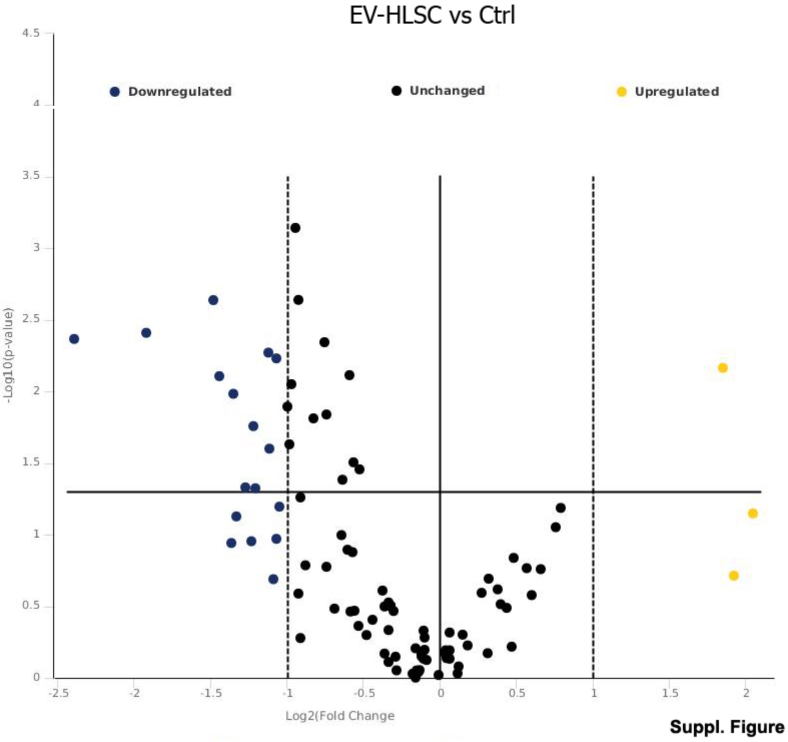

Suppl. Figure 3

Supplementary Figure 1: Effects of EV-HLSC administration on liver function and morphology in a murine model of NASH. Levels of aspartate aminotransferase (AST) (A), expressed as U/l, and albumin (B), expressed as g/dL, in serum of control mice (CTRL) and of mice fed with MCDD for 4 weeks treated with vehicle alone (NASH) or with iv injection of different doses of EVs (Dose 1: 5x109 EVs/mouse/injection, n=9; Dose 2: 2.5x109 EVs/mouse/injection, n=9 and Dose 3: 2.5x108 EVs/mouse/injection, n=8) and sacrificed at week 4. C) Histological quantification of the surface area occupied by steatosis vacuoles in MCDD-fed mice injected with different dose of EVHLSCs or with vehicle alone (NASH). Data shown for AST, Albumin and steatosis represents mean±SD. No statistical significant differences have been observed performing Anova with NewmanKeuls multicomparison test.

Supplementary Figure 2. Bio-distribution of EV-HLSCs. Quantification of fluorescence intensity in lungs of NASH and healthy mice injected with fluorescent EV-HLSCs. **$p<0.01$ healthy control mice vs NASH mice, student’s T Test.

Supplementary Figure 3. The volcano plots identify significant changes in gene expression between EV-HLSC-treated NASH (EV-HLSC) mice respect to control healthy group (Ctrl). The volcano plot displays the statistical significance ($p<0.005$) versus fold-change on the y- and x-axes, respectively.

Supplementary Table1. Fold Change values of genes analysed using Mouse Fibrosis PCR Array

	NASH vs Ctrl			EV-HLSC vs NASH			EV-HLSC vs Ctrl		
**Symbol**	**Fold Change**	**95% CI**	**p-value**	**Fold Change**	**95% CI**	**p-value**	**Fold Change**	**95% CI**	**p-value**
**Acta2**	2.3173	(0.82. 3.81)	0.009777	0.5354	(0.36. 0.71)	0.007	1.2407	(0.38. 2.10)	0.67304
**Agt**	0.778	(0.54. 1.02)	0.162262	1.1354	(0.47. 1.80)	0.54849	0.8834	(0.40. 1.37)	0.93658
**Akt1**	1.0354	(0.78. 1.29)	0.900035	0.6183	(0.40. 0.84)	0.03639	0.6402	(0.38. 0.90)	0.1006
**Bcl2**	1.1766	(0.71. 1.64)	0.447135	0.3911	(0.23. 0.55)	0.01927	0.4602	(0.32. 0.60)	0.00535
**Bmp7**	0.1734	(0.11. 0.24)	0.003927	1.1006	(0.80. 1.40)	0.52413	0.1908	(0.12. 0.26)	0.00429
**Cav1**	0.7828	(0.43. 1.13)	0.304835	0.941	(0.47. 1.42)	0.97574	0.7366	(0.32. 1.15)	0.39281
**Ccl11**	0.9932	(0.00001. 2.53)	0.951701	0.5349	(0.00001. 1.36)	0.52967	0.5312	(0.00001. 1.33)	0.52657
**Ccl12**	0.5895	(0.09. 1.08)	0.382763	0.8928	(0.00001. 2.00)	0.89343	0.5264	(0.01. 1.05)	0.25771
**Ccl3**	11.9747	(6.24. 17.71)	0.003656	0.3445	(0.10. 0.59)	0.04104	4.1258	(1.55. 6.70)	0.07098
**Ccr2**	7.6458	(5.83. 9.47)	0	0.2259	(0.13. 0.32)	1.1E-05	1.7269	(0.90. 2.55)	0.06502
**Cebpb**	0.5256	(0.31. 0.74)	0.041405	0.9202	(0.40. 1.44)	0.96215	0.4837	(0.20. 0.77)	0.06368
**Col1a2**	2.2113	(1.21. 3.21)	0.008617	0.2704	(0.11. 0.43)	0.00207	0.5979	(0.20. 0.99)	0.16768
**Col3a1**	1.9321	(1.03. 2.84)	0.021212	0.2469	(0.06. 0.43)	0.00173	0.4769	(0.08. 0.87)	0.10693
**Ctgf**	0.9463	(0.18. 1.72)	0.695945	0.7117	(0.06. 1.37)	0.42241	0.6735	(0.35. 0.99)	0.13247
**Cxcr4**	7.1934	(3.61. 10.78)	0.00001	0.5021	(0.34. 0.66)	0.00255	3.6117	(1.53. 5.69)	0.00683
**Dcn**	1.1346	(1.05. 1.22)	0.02177	0.9218	(0.79. 1.05)	0.30223	1.0458	(0.91. 1.18)	0.48205
**Edn1**	1.6794	(0.51. 2.85)	0.215387	0.5342	(0.25. 0.82)	0.06204	0.8971	(0.33. 1.46)	0.62207
**Egf**	0.6398	(0.41. 0.87)	0.0566	0.6722	(0.33. 1.01)	0.20214	0.4301	(0.20. 0.66)	0.01744
**Eng**	0.7227	(0.62. 0.82)	0.003473	0.5436	(0.24. 0.85)	0.112	0.3929	(0.18. 0.61)	0.01037
**Fasl**	5.3126	(1.94. 8.68)	0.030631	0.255	(0.09. 0.42)	0.04226	1.3549	(0.60. 2.11)	0.32428
**Grem1**	0.5758	(0.19. 0.97)	0.246986	1.3863	(0.52. 2.26)	0.57727	0.7982	(0.48. 1.11)	0.31432
**Hgf**	1.2463	(0.86. 1.63)	0.21641	0.6516	(0.37. 0.94)	0.11709	0.8121	(0.53. 1.10)	0.34099
**Ifng**	1.125	(0.08. 2.17)	0.431795	0.7303	(0.00001. 1.51)	0.47898	0.8215	(0.23. 1.41)	0.887
**Il10**	0.9133	(0.10. 1.72)	0.802507	1.19	(0.21. 2.17)	0.85417	1.0869	(0.72. 1.46)	0.83257
**Il13**	0.4836	(0.30. 0.67)	0.028099	1.6506	(1.19. 2.11)	0.02312	0.7982	(0.48. 1.11)	0.31432
**Il13ra2**	1.3238	(0.00001. 2.65)	0.95142	1.0477	(0.00001. 2.12)	0.55665	1.3869	(0.00001. 3.31)	0.60546
**Il1a**	1.1588	(0.73. 1.59)	0.416864	0.5541	(0.28. 0.83)	0.09484	0.6421	(0.42. 0.86)	0.04129
**Il1b**	1.9201	(0.96. 2.88)	0.098822	0.4654	(0.12. 0.81)	0.13233	0.8936	(0.38. 1.41)	0.99589
**Il4**	0.7353	(0.34. 1.13)	0.389147	1.5395	(0.35. 2.73)	0.35135	1.132	(0.30. 1.96)	0.59327
**Il5**	0.6931	(0.35. 1.04)	0.222117	1.1517	(0.66. 1.64)	0.63656	0.7982	(0.48. 1.11)	0.31432
**Ilk**	0.6871	(0.62. 0.75)	0.000159	0.9658	(0.73. 1.20)	0.89094	0.6636	(0.51. 0.81)	0.00768
**Inhbe**	1.2968	(1.10. 1.49)	0.011228	0.3934	(0.22. 0.56)	0.00046	0.5101	(0.28. 0.74)	0.00888
**Itga1**	0.9731	(0.78. 1.16)	0.831254	0.5598	(0.20. 0.91)	0.19797	0.5447	(0.20. 0.89)	0.16362
**Itga2**	1.4334	(0.37. 2.50)	0.348073	0.4647	(0.11. 0.82)	0.11441	0.666	(0.16. 1.17)	0.34357
**Itga3**	1.1982	(1.06. 1.34)	0.022281	0.3318	(0.08. 0.59)	0.02319	0.3975	(0.09. 0.70)	0.07446
**Itgav**	1.1242	(0.86. 1.38)	0.32439	0.4736	(0.21. 0.73)	0.03828	0.5324	(0.26. 0.81)	0.05489
**Itgb1**	0.867	(0.73. 1.01)	0.140229	0.6905	(0.46. 0.92)	0.07516	0.5987	(0.41. 0.79)	0.01447
**Itgb3**	1.5183	(0.92. 2.12)	0.064605	0.5133	(0.15. 0.88)	0.09911	0.7794	(0.31. 1.25)	0.67665
**Itgb5**	0.9778	(0.78. 1.17)	0.9253	0.6058	(0.42. 0.79)	0.02376	0.5924	(0.44. 0.74)	0.00452
**Itgb6**	0.5635	(0.20. 0.93)	0.188038	1.4054	(0.57. 2.24)	0.48223	0.7919	(0.48. 1.10)	0.29715
**Itgb8**	1.0003	(0.63. 1.37)	0.979587	1.0439	(0.34. 1.75)	0.65543	1.0442	(0.35. 1.74)	0.64171
**Jun**	1.512	(0.86. 2.16)	0.148051	0.5138	(0.40. 0.63)	0.00064	0.7768	(0.42. 1.13)	0.31744
**Lox**	6.2362	(4.07. 8.41)	0.000315	0.1649	(0.04. 0.28)	0.00072	1.0282	(0.28. 1.77)	0.64166

Ltbp1	2.3205	(1.45. 3.19)	0.009011	0.4774	(0.29. 0.67)	0.01459	1.1078	(0.80. 1.42)	0.49886
Mmp13	6.3838	(2.86. 9.90)	0.018047	0.189	(0.08. 0.29)	0.02081	1.2066	(0.84. 1.57)	0.25484
Mmp14	2.4819	(1.97. 2.99)	0.000535	0.3673	(0.14. 0.60)	0.015	0.9116	(0.36. 1.47)	0.88269
Mmp1a	6.9291	(4.95. 8.91)	0.000065	0.2276	(0.11. 0.35)	0.00042	1.577	(0.73. 2.43)	0.17395
Mmp2	2.2953	(1.30. 3.29)	0.004088	0.6459	(0.42. 0.88)	0.04072	1.4826	(0.73. 2.23)	0.17137
Mmp3	1.602	(0.34. 2.86)	0.314204	0.4466	(0.07. 0.82)	0.19749	0.7154	(0.20. 1.23)	0.50196
Mmp8	3.4484	(0.61. 6.29)	0.149496	0.3765	(0.04. 0.71)	0.19082	1.2983	(0.74. 1.86)	0.2407
Mmp9	0.9057	(0.00001. 2.22)	0.63655	0.519	(0.00001. 1.25)	0.35808	0.4701	(0.10. 0.84)	0.20458
Myc	1.3872	(0.00001. 3.21)	0.720767	0.4902	(0.27. 0.71)	0.02042	0.68	(0.00001. 1.59)	0.33985
Nfkb1	1.0404	(0.83. 1.25)	0.640613	0.4826	(0.27. 0.70)	0.01916	0.5021	(0.30. 0.71)	0.01274
Pdgfa	2.3865	(1.67. 3.10)	0.005366	0.5858	(0.36. 0.81)	0.05099	1.398	(0.92. 1.88)	0.14522
Pdgfb	2.7182	(1.43. 4.01)	0.025223	0.2914	(0.08. 0.50)	0.03021	0.792	(0.32. 1.26)	0.77502
Plat	6.9183	(0.90. 12.94)	0.003338	0.1569	(0.06. 0.26)	0.00275	1.0857	(0.00. 2.17)	0.93073
Plau	4.1265	(2.44. 5.81)	0.004185	0.2276	(0.04. 0.42)	0.00908	0.9391	(0.21. 1.67)	0.75017
Plg	0.3715	(0.33. 0.41)	0.000001	0.9641	(0.48. 1.45)	0.83407	0.3582	(0.18. 0.54)	0.0023
Serpina1 a	0.2375	(0.15. 0.32)	0.000049	2.1905	(1.35. 3.03)	0.00608	0.5202	(0.41. 0.63)	0.00072
Serpine1	3.927	(1.61. 6.24)	0.019076	0.4304	(0.19. 0.68)	0.05025	1.6902	(0.86. 2.52)	0.08861
Serpinh1	1.3918	(1.01. 1.78)	0.06524	0.7352	(0.30. 1.17)	0.37712	1.0232	(0.45. 1.60)	0.66888
Smad2	0.8425	(0.63. 1.06)	0.232039	0.8229	(0.57. 1.08)	0.25449	0.6932	(0.51. 0.88)	0.03494
Smad3	0.7426	(0.57. 0.92)	0.041624	0.4947	(0.19. 0.80)	0.09233	0.3673	(0.15. 0.58)	0.00782
Smad4	0.7271	(0.56. 0.89)	0.023957	0.9296	(0.62. 1.24)	0.73963	0.6759	(0.49. 0.86)	0.03122
Smad6	1.1697	(0.90. 1.44)	0.246943	0.3315	(0.00001. 0.67)	0.04746	0.3877	(0.00001. 0.79)	0.11436
Smad7	0.8074	(0.52. 1.09)	0.308004	0.5265	(0.01. 1.04)	0.33705	0.4251	(0.03. 0.82)	0.11116
Snai1	1.675	(0.00001. 3.65)	0.85244	0.4124	(0.11. 0.71)	0.06901	0.6908	(0.00001. 1.48)	0.43384
Sp1	0.7739	(0.57. 0.98)	0.113732	0.5353	(0.18. 0.89)	0.2047	0.4143	(0.15. 0.68)	0.04669
Stat1	1.1085	(0.78. 1.44)	0.450519	0.5089	(0.31. 0.71)	0.0152	0.5642	(0.37. 0.76)	0.0154
Stat6	0.6947	(0.35. 1.04)	0.231383	0.3808	(0.19. 0.57)	0.02298	0.2645	(0.16. 0.37)	0.00389
Tgfb1	1.6919	(1.09. 2.30)	0.04588	0.311	(0.17. 0.45)	0.00823	0.5262	(0.37. 0.68)	0.00229
Tgfb2	1.1598	(0.53. 1.78)	0.457972	0.4105	(0.19. 0.63)	0.03432	0.4761	(0.34. 0.62)	0.00587
Tgfb3	2.1165	(1.21. 3.02)	0.002523	0.2045	(0.09. 0.32)	3.9E-05	0.4329	(0.13. 0.73)	0.04741
Tgfbr1	1.123	(0.82. 1.43)	0.533936	0.7069	(0.40. 1.02)	0.18515	0.7938	(0.41. 1.18)	0.46284
Tgfbr2	0.9967	(0.80. 1.20)	0.981178	0.5081	(0.28. 0.74)	0.02511	0.5064	(0.28. 0.73)	0.02334
Tgif1	1.6514	(1.25. 2.05)	0.012599	0.603	(0.43. 0.78)	0.01813	0.9958	(0.79. 1.20)	0.95521
Thbs1	3.5986	(1.72. 5.48)	0.008686	0.183	(0.09. 0.28)	0.00484	0.6585	(0.35. 0.96)	0.12748
Thbs2	2.7908	(1.44. 4.14)	0.001948	0.4711	(0.35. 0.59)	0.00223	1.3149	(0.69. 1.94)	0.30602
Timp1	12.3755	(0.00001. 27.25)	0.211976	0.3064	(0.00001. 0.78)	0.32557	3.7919	(0.00001. 7.76)	0.19331
Timp2	1.4423	(1.06. 1.82)	0.045588	0.6458	(0.46. 0.83)	0.03652	0.9315	(0.69. 1.17)	0.63683
Timp3	0.9086	(0.65. 1.17)	0.495181	0.6831	(0.25. 1.12)	0.4916	0.6207	(0.22. 1.02)	0.32832
Timp4	0.5591	(0.18. 0.93)	0.217489	1.382	(0.51. 2.26)	0.59346	0.7727	(0.48. 1.06)	0.24578
Tnf	6.2125	(3.48. 8.95)	0.002238	0.2443	(0.01. 0.48)	0.01523	1.5175	(0.07. 2.96)	0.2641
Vegfa	0.4725	(0.33. 0.62)	0.000808	0.9792	(0.40. 1.56)	0.86329	0.4627	(0.22. 0.70)	0.02507
Actb	1.3747	(1.27. 1.48)	0.00016	0.6744	(0.56. 0.79)	0.00165	0.9272	(0.76. 1.10)	0.46795
B2m	0.7518	(0.58. 0.93)	0.042485	1.6581	(0.97. 2.35)	0.042	1.2465	(0.77. 1.73)	0.20293
Gapdh	0.8799	(0.67. 1.09)	0.31751	1.1898	(0.95. 1.43)	0.15023	1.0469	(0.82. 1.28)	0.73474
Gusb	1.4665	(1.11. 1.83)	0.030327	0.6372	(0.47. 0.81)	0.02038	0.9344	(0.75. 1.12)	0.52235
Hsp90ab 1	0.8108	(0.70. 0.93)	0.030274	1.0082	(0.52. 1.50)	0.73206	0.8174	(0.41. 1.23)	0.7112

Supplementary Table 2: List of protein vehicled by EV-HLSCs

Protein name	Gene name	F.U. Mean	F.U. SD
ApoC3	APOC3	66202,7	2198,6
GM-CSF R alpha	CSF2RA	2148,9	2746,6
LAG-3	LAG3	1986,4	270,6
Amylin	IAPP	753,9	65,3
BD-1	DEFB1	713,6	25,3
HGFR	MET	655,6	98,2
GDF11	GDF11	653,6	21,6
MSHa	MSX1	653,4	84,2
GDF9	GDF9	643,9	4,0
GDF3	GDF3	607,3	35,4
IL-21	IL21	605,4	92,7
IL-13	IL13	603,4	169,1
Granzyme A	GZMA	587,5	44,2
GDF5	GDF5	583,8	37,4
GDF8	MSTN	576,7	21,0
GRO	CXCL2	549,2	97,9
IFN-gamma	IFNG	538,3	9,3
IL-7	IL7	500,6	70,7
IL-6	IL6	500,2	77,0
IL-1alpha	IL1A	491,2	52,7
IL-5	IL5	484,6	9,6
IL-8	CXCL8	479,0	50,9
CNTF	CNTF	464,9	2,5
IL-2	IL2	457,6	64,5
TNF-alpha	LTA	454,6	200,4
CD71	TFRC	447,4	109,3
GDF1	GDF1	436,5	3,8
BAI-1	ADGRB1	436,2	383,1
ErbB4	ERBB4	428,5	0,5
CNTF R alpha	CNTFR	422,6	15,0
Complement component C2	C2	416,2	63,7
Mammaglobin A	SCGB2A2	405,4	47,5
Clusterin	CLU	404,1	109,6
TGF-beta 1	TGFB1	403,2	178,5
ANG-1	ANGPT1	402,4	232,9
ENPP2	ENPP2	388,6	95,4
CD30	TNFRSF8	386,5	49,4
TNF-beta	LTB	385,5	179,6

MIP-3 beta / CCL19	CCL19	385,2	59,9
GFR alpha-3	GFRA3	371,9	63,4
IL-15	IL15	368,4	67,7
EDG-1	S1PR1	367,8	17,6
BMP-7	BMP7	349,7	55,0
LH	LHCGR	346,2	121,5
CTACK	CCL27	339,2	18,3
Insulysin / IDE	IDE	334,7	420,2
CA19-9	MUC16	333,1	56,1
NPTX1	NPTX1	331,4	57,2
GREMLIN	GREM1	330,1	14,2
IL-23	IL23A	327,8	406,7
CCL28	CCL28	326,5	6,1
C-peptide	INS	325,6	117,6
Carboxypeptidase N subunit 2/CPN2	CPN2	324,8	98,3
ACK1	TNK2	324,6	0,6
VDUP-1	TXNIP	320,2	201,6
Troponin C	TNNC1	319,7	99,4
Cytokeratin 19	KRT19	319,0	117,3
HCR / CRAM-A/B	CCHCR1	318,1	110,4
Aldolase C	ALDOC	315,6	32,3
Lyn	LYN	314,4	102,5
Growth Hormone (GH)	GH1	311,2	16,1
Insulin R	INSR	310,8	53,1
IL-2 R beta	IL2RB	309,3	18,8
CD 163	CD163	308,9	143,5
CHI3L1	CHI3L1	308,8	157,5
FER	FER	308,7	137,3
Alpha Lactalbumin	LALBA	308,3	11,9
IL-9	IL9	306,3	24,5
TRA-1-60	PODXL	305,0	171,0
IL-1 R6 / IL-1 Rrp2	IL1RL2	301,8	76,7
Dkk-4	DKK4	301,7	15,7
FGF-16	FGF16	301,2	28,8

ALPP (alkaline phosphatase, placental)	ALPP	300,3	54,7
IL-2 R gamma	IL2RG	300,2	15,6
MIP-1beta / CCL4	CCL4	296,3	97,4
MCP-1 / CCL2	CCL2	293,0	95,0
Creatinine	CSH1	292,3	82,3
AGRP	AGRP	290,8	46,7
Ceruloplasmin	CP	289,4	107,7
TRA-1-81	PODXL	288,9	164,3
Aldolase A	ALDOA	287,1	40,5
Glut5	SLC2A5	285,0	32,1
APOA4	APOA4	282,6	32,8
MINA	RIOX2	281,0	42,1
Tec	TEC	279,7	117,7
GITR L / TNFSF18 L	TNFSF18L	278,8	9,5
Endoglin / CD105	ENG	278,2	0,9
BTC	BTC	277,6	34,6
FGF-10 / KGF-2	FGF10	276,2	28,9
SERPING1/Plasma protease C1 inhibitor	SERPING1	273,0	154,4
Btk	BTK	272,5	76,5
IL-1 RI	IL1R1	270,7	31,2
MIG / CXCL9	CXCL9	268,1	46,6
MMP20	MMP20	267,5	10,3
MIP 2 / CXCL2	CXCL2	267,3	43,3
Kallikrein 2	KLK2	266,4	124,1
CTLA-4 / CD152	CTLA4	264,0	13,1
Ubiquitin+1	UBB	262,9	147,8
IL-10 R alpha	IL10RA	261,9	40,7
Cryptic	CFC1	261,3	0,1
FGF-18	FGF18	261,1	44,8
FRK	FRK	260,8	34,2
BMP-5	BMP5	259,1	9,9

Angiopoietin-like 1	ANGPTL1	258,8	32,7
Alpha 1-Acid Glycoprotein/ORM	ORM1	257,7	26,1
PDGF R beta	PDGFRB	256,3	65,4
Follistatin	FST	253,4	24,4
GLP-1	ZGLP1	252,8	88,8
P-selectin	SELP	250,0	104,2
BNP	NPPB	248,7	27,0
Coagulation factor XIII A	F13A1	247,8	46,0
TXK	TXK	245,4	69,2
CBP /KAT3A	CREBBP	244,9	39,8
PARC / CCL18	CCL18	243,5	45,0
Angiostatin	PLG	243,5	20,5
EphA4	EPHA4	242,6	18,0
Ntn1	NTN1	242,1	111,4
Thrombospondin-2	THBS2	241,8	115,7
Chordin-Like 2	CHRDL2	240,3	19,6
IL-1 R4 /ST2	IL1RL1	239,6	9,9
Corticosteroid-binding globulin	SERPINA6	236,8	40,9
IL-11	IL11	235,7	10,8
Frizzled-1	FZD1	235,2	7,1
IL-17	IL17A	233,6	24,0
S100 A8/A9	S100A8	231,2	26,7
MMP-8	MMP8	231,0	89,6
Itk	ITK	230,7	33,5
MATK	MATK	229,8	76,5
PR Isoform B/NR3C3	PGR	229,6	65,8
SHBG	SHBG	228,5	135,7
LTF	LTF	228,5	13,5
IL-23p19	IL23A	228,1	111,3
IL-17RD	IL17RD	228,0	148,4
Galanin	GAL	226,6	95,8
BMP-8	BMP8B	223,5	11,3
L-selectin / CD62L	SELL	223,0	25,9
BNIP2	BNIP2	222,2	61,6
LRG1	LRG1	221,7	132,9
CV-2 / Crossveinless-2	BMPER	221,0	35,3
TRAIL R1	TNFRSF10A	220,2	88,1
FGF-11	FGF11	218,2	14,5

G-CSF	CSF3	218,1	24,0
Fyn	FYN	218,1	101,2
PEPSINOGEN I	PGC	217,9	66,2
Fibrinopeptide A	FGA	214,3	76,4
FGF-17	FGF17	214,0	13,0
Glut1	SLC2A1	213,9	31,5
GASP-2 / WFIKKN	WFIKKN1	211,8	2,4
Frizzled-3	FZD3	210,6	2,4
ACTH	POMC	210,3	20,2
SAA	SAA1	209,9	71,3
TYRO10	DDR2	209,7	115,3
TRAIL R2	TNFRSF10B	207,0	56,5
Sonic Hedgehog (Shh N-terminal)	SHH	205,2	43,1
ZAP70	ZAP70	204,9	45,6
GADD45A	GADD45A	204,3	96,5
NF1	NF1	202,9	89,6
SDF-1	CXCL12	201,7	15,9
Hepassocin	FGL1	200,2	1,4
LPS	IRF6	198,6	77,2
IL-13 R alpha 1	IL13RA1	197,6	24,0
CCL14 / HCC-1 / HCC-3	CCL14	197,6	15,3
IL-20 R alpha	IL20RA	197,2	21,0
Glut2	SLC2A2	197,1	12,4
MSP alpha-chain / MST1	MST1	196,4	70,8
SLPI	SLPI	195,4	97,7
IL-1 F10 / IL-1HY2	IL1F10	194,1	27,5
PI 3Kinase p85 beta	PIK3R1	193,8	61,3
MAC-1	ITGAM	193,3	41,7
TGF-beta 5	TGFB5	192,7	58,6
CA 125	MUC16	191,6	52,6
PDGF R alpha	PDGFRA	191,5	38,4
Calbindin	CALB1	191,5	46,9
AFP	AFP	191,4	41,9
PAI-1	SERPINE1	191,1	154,1
Netrin G2	NTNG2	190,7	61,2
FAM3B	FAM3B	190,7	12,8

hCG alpha	CGA	190,4	86,6
BAX	BAX	190,2	27,4
ADAMTS-17	ADAMTS17	190,1	4,1
GRP	GRP	189,1	98,1
Activin C	IHBC	188,9	76,3
LCK	LCK	187,3	8,7
HP/Haptoglobin	HP	186,2	78,6
FGF-13 1B	FGF13	186,2	13,0
IL-1 R8 / IL1RAPL1	IL1RAPL1	185,9	26,7
EV15L	EVI5L	185,3	70,3
CK-MB	CKB	185,3	3,9
PPARg2/NR1C3	PPARG	184,3	41,6
HSP27	HSPB1	183,6	28,0
FGF-5	FGF5	183,5	22,2
ASPH	ASPH	182,9	35,5
Fibronectin	FN1	181,9	105,3
FGF-20	FGF20	181,5	11,9
GASP-1 / WFIKKNRP	WFIKKN2	181,3	8,5
APC	APC	181,0	13,9
VGF/Neurosecretory protein VGF	VGF	180,5	83,8
MUSK	MUSK	179,3	66,6
Ferritin	FTL	178,3	87,6
FGFR1 alpha	FGFR1	178,0	70,4
CNDP1	CNDP1	177,9	36,0
XEDAR / TNFRSF27	EDA2R	177,4	84,6
Integrin alpha V	ITGAV	177,2	54,6
ABL1	ABL1	176,9	6,7
CD97	ADGRE5	176,6	7,6
EMAP-II / EML2	EML2	175,7	1,7
IL-17B R	IL17RB	175,2	46,9
Thyroid Peroxidase / TPX	TPO	173,7	84,8
BCAM	bcam	173,6	33,0
GM-CSF	CSF2	173,3	16,6
GMNN	GMNN	172,8	48,4
DR3 / TNFRSF25	TNFRSF25	171,2	4,2
PPP2R5C	PPP2R5C	170,7	62,3
CLC	CLC	169,9	19,8
LDL R	LDLR	167,9	59,7
p21	CDKN1A	167,2	47,5

TACI / TNFRSF13B	TNFRSF13B	166,8	69,7
Wilms Tumor 1	WT1	166,8	114,9
NRG3	NRG3	166,8	58,8
E-selectin	SELE	165,8	11,8
CRTH-2	PTGDR2	165,2	19,6
EphA3	EPHA3	164,8	30,2
TARC / CCL17	CCL17	164,2	84,4
FOXN3	FOXN3	164,0	38,7
Endostatin	COL18A1	163,8	7,7
ANGPTL3	ANGPTL3	163,7	67,8
TSH	TSHB	163,1	92,1
Calcitonin	CALCA	162,7	75,7
EphA6	EPHA6	160,6	20,0
Kremen-1	KREMEN1	160,5	26,9
Glut3	SLC2A3	159,7	2,6
Marapsin/Pancreasin	PRSS27	159,2	70,5
MMP-2	MMP2	158,6	56,5
hCGb	CGB3	158,3	78,3
EphA1	EPHA1	158,0	73,0
GSR (Glutathione Reductase)	GSR	157,8	65,1
FIH (Calcium Sensing Receptor)	CASR	157,4	66,5
ACE/CD143	ACE	156,9	54,6
Complement component C9	C9	155,4	39,9
Neurokinin-A	TAC1	154,4	58,8
CD40	CD40	154,3	6,6
CD59 complement fragment	CD59	153,6	38,0
HE4	WFDC2	152,2	64,1
ADAMTS-19	ADAMTS19	151,5	51,9
11b-HSD1	HSD11B1	151,4	29,3
FSH	FSHB	151,3	39,0
EphB3	EPHB3	150,9	90,3
Hepcidin	HAMP	150,3	12,4
Hck	HCK	150,1	1,7
OX40 Ligand / TNFSF4	TNFSF4	149,9	123,5
IL-10	IL10	149,8	7,4
