# Supplementary material for: HLSC-Derived Extracellular Vesicles Attenuate Liver Fibrosis and Inflammation in a Murine Model of Non-alcoholic Steatohepatitis
Source: Mol Ther. 2019 Oct 28;28(2):479–89. doi: 10.1016/j.ymthe.2019.10.016 (PMC7001005; doi:10.1016/j.ymthe.2019.10.016)
Supplement: Document S2. Article plus Supplemental Information [file mmc3.pdf]

Molecular Therapy
Original Article

HLSC-Derived Extracellular Vesicles Attenuate Liver Fibrosis and Inflammation in a Murine Model of Non-alcoholic Steatohepatitis

Stefania Bruno,[1,2] Chiara Pasquino,[1,2] Maria Beatriz Herrera Sanchez,[2,3] Marta Tapparo,[1,2] Federico Figliolini,[2,3] Cristina Grange,[1,2] Giulia Chiabotto,[1] Massimo Cedrino,[2] Maria Chiara Deregibus,[3] Ciro Tetta,[4] and Giovanni Camussi[1,2]

[1]Department of Medical Sciences, University of Torino, Torino, Italy; [2]Molecular Biotechnology Centre, University of Torino, Torino, Italy; [3]2i3T Società per la Gestione dell'Incubatore di Imprese e per il Trasferimento Tecnologico Scarl, University of Torino, Torino, Italy; [4]Unicyte Srl, Torino, Italy

Extracellular vesicles (EVs) are membrane vesicles released virtually by all cell types. Several studies have shown that stem cell-derived EVs may mimic both *in vitro* and *in vivo* the biological effects of the cells. We recently demonstrated that non-alcoholic steatohepatitis (NASH) is inhibited by treatment with human liver stem cells (HLSCs). The aim of the present study was to evaluate whether EVs released by HLSCs influence the progression of NASH, induced by a diet deprived of methionine and choline, in immunocompromised mice. EV treatment was initiated after 2 weeks of diet with a biweekly administration of three different doses. Bio-distribution evaluated by optical imaging showed a preferential accumulation in normal and, in particular, in fibrotic liver. EV treatment significantly improved liver function and reduced signs of liver fibrosis and inflammation at both morphological and molecular levels. In particular, we observed that, out of 29 fibrosis-associated genes upregulated in NASH liver, 28 were significantly downregulated by EV treatment. In conclusion, HLSC-derived EVs display anti-fibrotic and anti-inflammatory effects in a model of chronic liver disease, leading to an improvement of liver function.

INTRODUCTION

Non-alcoholic fatty liver disease (NAFLD) is one of the most common causes of chronic liver disease. NAFLD progression may lead to non-alcoholic steatohepatitis (NASH), characterized by liver inflammation and fibrosis leading to end-stage liver disease, including cirrhosis and hepatocellular carcinoma.[1,2] The establishment of anti-fibrotic and anti-inflammatory therapies is one of the major clinical requirement to impinge on liver fibrosis and, especially, NASH.

Human liver stem cells (HLSCs) are an easily obtainable and expandable stem cell population derived from human adult liver cells.[3,4] HLSCs share with mesenchymal stromal cells (MSCs) the expression of typical surface markers (CD73, CD29, CD105, CD90, and CD44), the differentiation capacity (osteoblasts), and the immunomodulatory properties.[3–5] Moreover, HLSCs show a specific hepatic commitment as they express typical markers of liver cells such as alpha-feto protein, albumin, and cytokeratins 8 and 18.[3,4] HLSCs are negative for the hematopoietic and cytokeratin 19 markers of oval cells and did not express *α-Sma*.[4] HLSCs are able to differentiate *in vitro* into functional hepatocytes and to form islet-like structures.[3,6,7] When tested *in vivo*, HLSCs are able to improve function and morphology in different experimental models of acute liver and renal injuries.[3,8,9] It has been recently demonstrated that HLSCs exhibit anti-fibrotic and anti-inflammatory effects in a murine model of NASH by regulating specific genes without the need for differentiation into mature hepatocytes.[4]

The aim of the present study was to investigate the mechanisms involved in the anti-fibrotic activity of HLSCs. Previous studies have shown that extracellular vesicles (EVs) are involved in the paracrine activity of HLSCs, including anti-fibrotic effects in pre-clinical models of chronic kidney diseases.[10,11] Cell-released EVs are a heterogeneous population of membrane vesicles, containing cytosol enclosed into a lipid bilayer. EVs may transfer their cargo (various types of RNAs, proteins, and bioactive lipids) from the producing cell to the recipient cell. EVs derived from different type of stem/progenitor cells may induce regenerative programs in injured organs/tissues by transcriptional or translational modification in injured target cells.[12,13]

To investigate whether HLSC-released EVs (EV-HLSCs) are implicated in the anti-fibrotic effect of HLSCs, we established a model of NASH induced by a methionine- and choline-deprived diet (MCDD) in immunodeficient mice and started a biweekly treatment with EVs after 2 weeks of diet. Mice were sacrificed at week 4; and functional, morphological, and molecular liver alterations were analyzed.

RESULTS

Characterization of EV-HLSCs

We evaluated EV expression markers using a recently described multiplex bead-based flow cytometry assay platform for EV

Received 12 August 2019; accepted 22 October 2019;
https://doi.org/10.1016/j.ymthe.2019.10.016.

Correspondence: Giovanni Camussi, Department of Medical Sciences, University of Torino, Corso Dogliotti 14, 10126 Torino, Italy.
E-mail: giovanni.camussi@unito.it

This is an open access article under the CC BY license (http://creativecommons.org/licenses/by/4.0/).

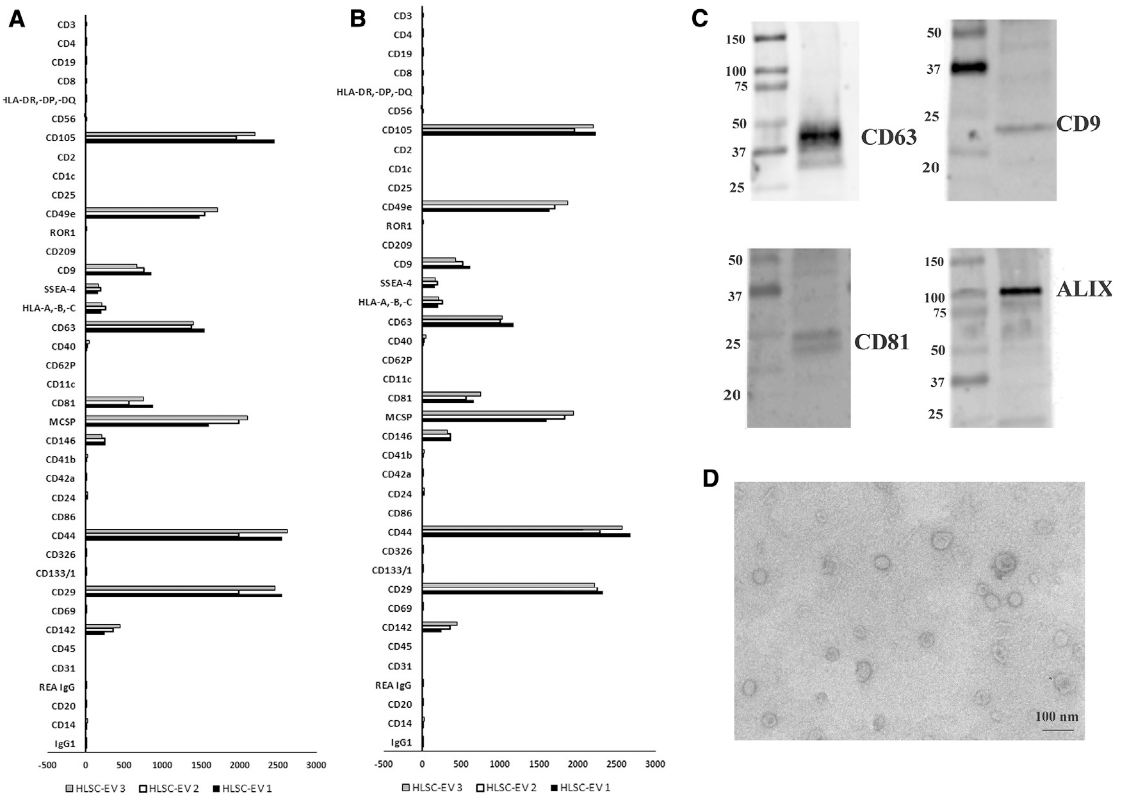

Figure 1. Characterization of EV-HLSCs

A multiplex bead-based flow cytometry assay was used to detect EV-HLSC surface signature. 39 multiplexed populations of dye-labeled antibody-coated capture beads were incubated with 3 different EV-HLSC samples. (A and B) Captured EV-HLSCs were counterstained with pan-tetraspanins APC-labeled (A) or with CD29 APC (B) detection antibodies. The graph shows a quantification of the median APC fluorescence values for all bead populations after background correction (medium control values subtracted from measured EV-HLSC values) of three different EV-HLSC preparations. No statistically significant differences were observed among the different EV-HLSC preparations evaluated. (C) Representative western blot analysis of exosomal markers (CD63, CD9, CD81, and Alix) in the EV-HLSCs. (D) Representative micrograph of transmission electron microscopy of EV-HLSCs. EVs were negatively stained with NanoVan (scale bar, 100 nm; magnification, 100,000×).

characterization.[14,15] This assay comprises 39 hard-dried capture bead populations, each of them coated with different monoclonal antibodies against 37 surface antigens and with two isotypic controls. Samples were detected by counterstaining with allophycocyanin (APC)-labeled detection antibodies against specific EV surface markers, such as tetraspanins (CD9, CD63, and CD81). EVs reacted with beads expressing CD9, CD63, and CD81. EVs also expressed high fluorescence intensity for CD29, CD44, CD105, and CD49e. Other markers detected at intermediate to low-positive fluorescence intensity comprised mainly CD142, CD146, SSEA-4, and MCSP (Figure 1). Hematopoietic (CD3, CD4, CD8, CD19, etc.), endothelial (CD31), and epithelial (CD326) markers were not detected in EVs (Figure 1A). When using APC-conjugated anti-CD29 antibody for detection in the same samples, a co-expression of tetraspanin exosomal markers, MSC markers CD29, CD44, CD105, CD49e, CD142, CD146, SSEA-4, and the adhesion molecule MCSP (Figure 1B) was observed. The expression of tetraspanins was confirmed by western blot analysis (Figure 1C).

By transmission electron microscopy, EV preparations showed a homogeneous pattern of nano-sized membrane vesicles (Figure 1D).

EV-HLSCs Improve Liver Function and Morphology of NASH Mice

To evaluate the potential therapeutic ability of EV-HLSCs on liver fibrosis and inflammation, we induced NASH by feeding severe combined immunodeficient (SCID) mice with an MCDD for 4 weeks, as described previously.[4,16–19] Different doses of EVs were administered through intravenous (i.v.) injection twice a week, starting at week 2 of the MCDD (Figure 2A).

Injection of doses 1 and 2 (dose 1: 5×10^9 EVs per mouse per injection; dose 2: 2.5×10^9 EVs per mouse per injection) of EVs induced a significant reduction of alanine aminotransferase (ALT) in the plasma of NASH mice (Figure 2B). Instead, the reduction of aspartate aminotransferase (AST) did not reach the statistical significance (Figure S1A). Serum albumin levels were increased in NASH mice treated with doses 1 and 2 of EVs, compared to NASH mice treated with vehicle alone (Figure S1B). The lower dose tested (dose 3: 2.5×10^8 EVs per mouse per injection) did not improve liver function in NASH-treated mice (Figure 2B; Figure S1). The effect of EVs on liver function, however,

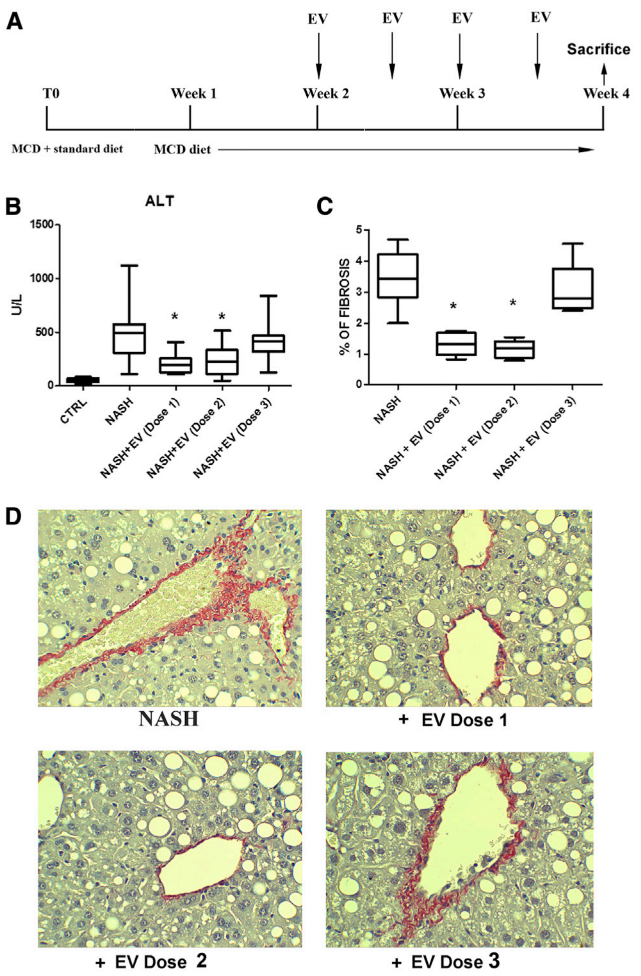

Figure 2. Effect of EV-HLSCs on Liver Function and Morphology

(A) Schematic representation of the experimental design to test EV-HLSCs in NASH mice, showing the weeks of feeding with MCCD, of EV-HLSC administration, and of sacrifice. (B) ALT levels expressed as units per liter were measured as a bio-marker of liver cell injury in serum of control mice (CTRL) and of mice fed with MCDD for 4 weeks treated with vehicle alone (NASH) or with iv injection of different doses of EVs (dose 1: 5×10^9 EVs per mouse per injection, n = 9; dose 2: 2.5×10^9 EVs per mouse per injection, n = 9; and dose 3: 2.5×10^8 EVs per mouse per injection, n = 8) and sacrificed at week 4. (C) Histological quantification of fibrosis in MCDD-fed mice injected with different doses of EVs or with vehicle alone (NASH) and sacrificed at week 4 by multiphase image analyses of 10 fields per section (original magnification, 400×). Data shown for ALT and fibrosis represent mean + SD. ANOVA with Newman-Keuls multicomparison test was performed. *$p < 0.05$, NASH mice injected with doses 1 and 2 of EV-HLSCs versus NASH mice treated with vehicle alone. (D) Representative light microscopy micrographs of liver histology of mice at week 4 of MCDD, treated with injection i.v. of different doses of EV-HLSCs or with vehicle alone (NASH) (original magnification, 400×). Sirius Red staining showed fibrosis in the peri-venous area of NASH mice. Red stain represents collagen fibers considered to be a marker of liver fibrosis.

was less effective than the one observed with HLSC treatment,[4] probably because the functional contribution of the liver engrafted HLSCs.

Liver fibrosis, as demonstrated by Sirius Red staining, showed an increase in NASH mice in both peri-venous and peri-portal areas (Figure 2D). Fibrosis was significantly reduced by treatment with doses 1 and 2 but not with dose 3 of EVs (Figure 2C). Treatment with EVs did not attenuate steatosis (Figure S1C).

Bio-distribution of EVs was performed by i.v. injection of labeled EVs in healthy and NASH mice. The organ uptake was assessed 3 h after administration. Accumulation of EVs was most prominent in the liver and at a low level in lungs, whereas no signal was observed in spleen and kidney (Figure 3A). Comparing the localization of fluorescent EVs in liver of NASH versus control healthy mice, we observed a significant accumulation of fluorescent EVs in the fibrotic liver (Figures 3B and 3C). Moreover, in comparing the fluorescent signaling in lungs of NASH versus control healthy mice, a significant lower accumulation of fluorescent EVs was observed in NASH mice (Figure S2). This could be due to an enhanced liver uptake of EVs in NASH mice.

EV-HLSC Treatment Downregulates Pro-fibrotic and Pro-inflammatory Genes in the Liver of NASH Mice

We investigated the possibility that the improvement in liver function and morphology after EV treatments may depend on the regulation of genes that are associated with the induction of fibrosis. To this purpose, liver tissue obtained from NASH mice, treated or not treated with dose 2 of EVs, was subjected to RNA isolation, and the Mouse Fibrosis RT^2 Profiler PCR Array was performed. The gene expression profiles of the healthy mice and NASH mice treated or not treated with EVs were compared. Figure 4A shows the heatmap of genes expressed in the experimental groups. Clustering analyses revealed significant changes in the expression of genes in NASH mice. Twenty-nine mRNAs out of 84 key genes were upregulated and 9 were downregulated in NASH mice compared to those from the healthy group (Figure 4B; Table S1). The majority of these genes encoded extracellular matrix (ECM) remodeling enzymes, transforming growth factor beta 1 (*Tgf-β1*) signaling molecules, and inflammatory cytokines involved in the development of liver fibrosis (Figure 4A). Comparing to NASH mice, 53 genes were downregulated by EV-HLSC treatment (Figure 4C; Table S1). Interestingly, 28 out of 29 mRNAs upregulated in NASH mice were significantly reverted by EV treatment (Figure 4D). The list of reverted genes included alpha smooth muscle actin (*α-Sma*), Collagen 1a1 (*Col1α1*), *Tgf-β1*, and the gene latent-transforming growth factor beta-binding protein 1 (*Ltbp1*). All these genes are known to be involved in fibrosis. EV treatment also modulated genes involved in tissue remodeling and in inflammation, such as TIMP metallopeptidase inhibitor 1 (*Timp-1*), matrix metallopeptidases (*Mmp-1a*, *-13*, *-14*, and *-8*), tumor necrosis factor alpha (*Tnf*), and interleukin-1 beta (*IL-1β*) (Figure 4D).

Real-time PCR confirmed that NASH mice had significantly upregulated levels of the pro-fibrotic genes *α-Sma*, *Col1a1* and *Tgf-β1* (Figure 5A) and of the pro-inflammatory genes *Tnf*, *IL-1β*, and interferon gamma (*Ifn-γ*) (Figure 5B). NASH mice treated with EVs had a significant reduction in the expression levels of all fibrotic and pro-inflammatory genes tested (Figures 5A and 5B). The anti-inflammatory

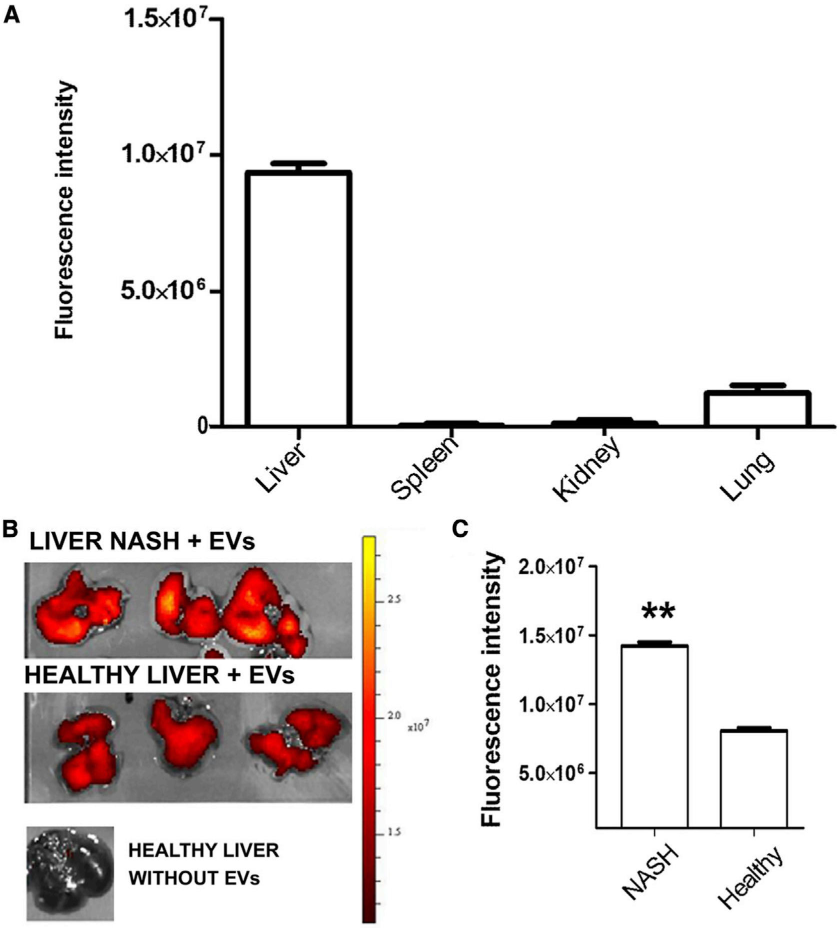

Figure 3. Bio-distribution of EV-HLSCs

(A) Quantification of fluorescence intensity in dissected organs of MCDD-fed mice, measured as average radiance ± SD at 3 h post-EV-HLSC administration (n = 6). (B) Representative images obtained by optical imaging of livers of NASH and healthy mice, collected 3 h after EV-HLSC or vehicle administration. (C) Quantification of fluorescence intensity in livers of NASH and healthy mice injected with fluorescent EV-HLSCs. **$p < 0.01$, NASH mice versus healthy control mice, Student's t test.

effect of EV treatment was also indicated by the reduction of inflammatory cells accumulated in the liver, as seen by immunofluorescence staining with the leucocyte marker CD45. Whereas inflammatory infiltrates were present in the liver of NASH mice, almost no leukocytes were detected in mice treated with EVs (Figure 5C). The increase of interleukin 10 (*IL-10*) expression level in NASH mice treated with EVs confirmed the anti-inflammatory effect of EVs (Figure 5D).

EV-HLSC Protein Cargo May Modulate Inflammation

Protein analysis showed the presence of 251 proteins in EVs (Table S2). Most of the molecules identified are cytokines and growth factors. Moreover, among the enriched EV proteins, we observed the presence of transmembrane tyrosine kinase receptors, MMPs, some enzymes (glutatione reductase, creatine kinase, and aspartate beta-hydroxylase), and transcription factors. Pathway enrichment analysis showed that the most enriched pathways were related to cytokine and inflammatory response, IL-10 anti-inflammatory activity, phosphatidylinositol 3-kinase (PI3K) pathways, and the p53 pathway (Figure 6; Table S3).

DISCUSSION

Results of the present study show that EV-HLSCs exerted beneficial effects on liver morphology, ameliorating fibrosis and inflammation by reprogramming liver gene expression. Previous studies have shown that EVs may induce epigenetic changes into recipient cells by transferring different molecules from the originator cells. It has been therefore suggested that stem cell-derived EVs may be exploited to promote tissue regeneration. Previous studies demonstrated that EVs obtained by MSCs of different origin improve acute liver injuries and increase survival in different lethal models of liver damages.[20,21] Moreover, MSC-derived EVs were shown to ameliorate hepatic ischemia reperfusion injury by modulating the inflammatory response[22,23] and by improving hepatic oxidant injury in toxic[24,25] and ischemic[26] models. EVs derived from different stem cells were also studied in different pre-clinical models of chronic liver injuries. MSC-derived EVs were able to reduce fibrosis in CCl4- and thioacetamide-induced chronic liver injures by alleviating hepatic inflammation and collagen deposition and by inhibiting epithelial-to-mesenchymal transition.[27–30] Moreover, EVs derived from serum reduced liver fibrosis in mice treated with CCl4 or thioacetic acid by improving liver function, reducing hepatocyte apoptosis, and suppressing inflammation.[31] These recent papers indicate that EVs can be exploited for therapy in liver diseases. EVs show a superior safety profile to cell-based therapy, as they pass biological barriers and act as effective transporters of different molecules.[32] Interestingly, EVs derived from HLSCs preferentially localized in liver—in particular, in fibrotic areas after systemic administration, as seen by optical imaging (OI). Different amounts of EV-HLSCs have been tested in NASH mice. We found that 2.5×10^9 EVs, injected twice a week starting at week 2 of an MCDD, are sufficient to obtain amelioration of liver function and morphology and a significant reduction of the percentage of fibrosis. At the molecular level, we found that 29 genes related to fibrosis and inflammation were upregulated in the liver of NASH mice, including transcripts for ECM remodeling enzymes (*mmp-1a*, *-3*, *-8*, *-14*, and *Timp-1*), *Tgf-β* and *Tgf-β* signaling molecules, inflammatory cytokines (*IL-1β* and *Tnf-α*) and chemokines (*Ccl3* and *Ccl2*), and chemokine receptor (*CXCR4*). In the liver of NASH mice treated with EVs, 28 out of 29 pro-fibrotic genes were downregulated. Notably, the mRNA expression level of lysyl oxidase (*Lox*), which is considered a serum biomarker of liver fibrosis in

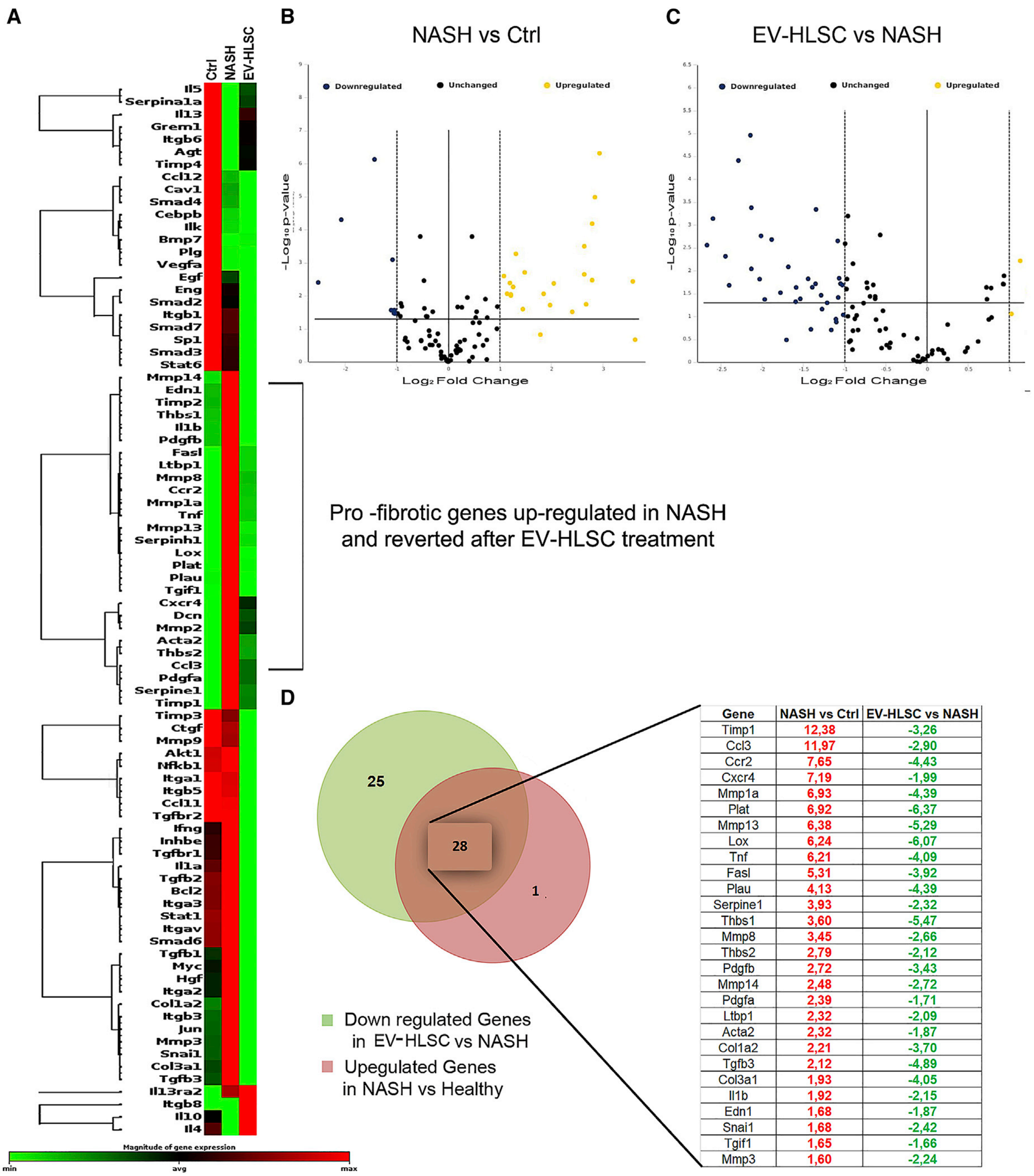

Gene	NASH vs Ctrl	EV-HLSC vs NASH
Timp1	12,38	-3,26
Ccl3	11,97	-2,90
Ccr2	7,65	-4,43
Cxcr4	7,19	-1,99
Mmp1a	6,93	-4,39
Plat	6,92	-6,37
Mmp13	6,38	-5,29
Lox	6,24	-6,07
Tnf	6,21	-4,09
Fasl	5,31	-3,92
Plau	4,13	-4,39
Serpine1	3,93	-2,32
Thbs1	3,60	-5,47
Mmp8	3,45	-2,66
Thbs2	2,79	-2,12
Pdgfb	2,72	-3,43
Mmp14	2,48	-2,72
Pdgfa	2,39	-1,71
Ltbp1	2,32	-2,09
Acta2	2,32	-1,87
Col1a2	2,21	-3,70
Tgfb3	2,12	-4,89
Col3a1	1,93	-4,05
Il1b	1,92	-2,15
Edn1	1,68	-1,87
Snai1	1,68	-2,42
Tgif1	1,65	-1,66
Mmp3	1,60	-2,24

Figure 4. Expression of Fibrosis-Related Genes in NASH Mice Treated or Not with EV-HLSCs Analyzed through a Fibrosis RT2 Profiler PCR Array (QIAGEN)
(A) Heatmap displaying hierarchical clustering of the entire dataset of expressed genes and indicating co-regulated genes across three different experimental groups: healthy (CTRL) mice, NASH mice, and EV-HLSC-treated mice. Clustering analysis showed that genes upregulated in NASH mice belong to pro-fibrotic and pro-inflammatory pathways; EV-HLSC treatment was able to restore their expression to healthy levels ($n = 4$ mice analyzed for each experimental condition). (B and C) The volcano plots identify significant changes in gene expression between groups: increased number of upregulated genes in NASH mice compared to healthy mice (B) and evident downregulation of gene expression in EV-HLSC-treated mice compared to the NASH group (C). The volcano plot displays the statistical significance ($p < 0.005$) versus fold change on the y and x axes, respectively. (D) Venn diagram and list of pro-fibrotic and pro-inflammatory genes regulated in NASH mice treated or not treated with HLSC EVs. The expression levels of genes are presented as fold regulation values (those greater than 1.6 are indicated in red, and those less than −1.6 are indicated in green) for NASH mice compared to those for controls (NASH vs Ctrl column) and for treated mice compared to those for the NASH group (EV-HLSC vs NASH column). Complete data are provided in Table S1.

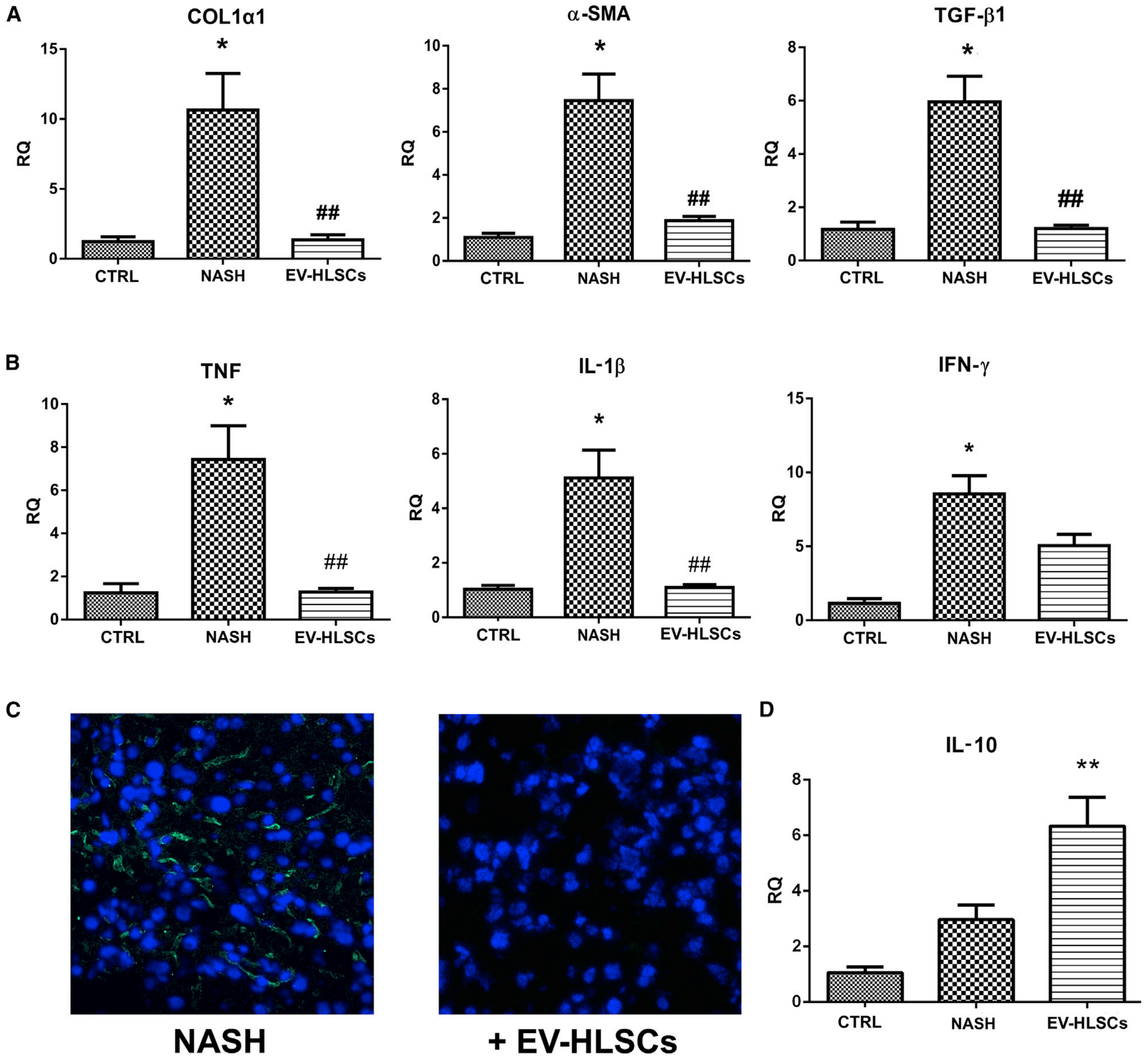

Figure 5. Effect of EV-HLSCs on Liver Fibrosis and Inflammation

(A) Gene expression levels of fibrotic markers (*Col1α1*, *α-Sma*, and *Tgf-β1*) in livers of healthy mice (CTRL) and of NASH mice injected with dose 2 of EV-HLSCs (EV-HLSC) or with vehicle alone (NASH). *$p < 0.05$, NASH mice treated with vehicle (NASH) versus normal healthy mice (CTRL); ##$p > 0.01$, NASH mice treated with vesicles (EV-HLSCs) versus NASH mice injected with vehicle alone (NASH). (B) Gene expression levels of pro-inflammatory cytokines (*Tnf, IL-1β* and *Ifn-γ*) in livers of healthy mice (CTRL) and of NASH mice treated with dose 2 of EV-HLSCs (EV-HLSCs) or with vehicle alone (NASH). For fibrotic and pro-inflammatory genes, data are expressed as relative quantification using the ΔΔCt method. Normalization was made using GAPDH as a housekeeping gene. An ANOVA with Newman-Keuls multicomparison test was performed. (C) Representative micrographs of liver cryosections from NASH mice injected or not injected with dose 2 of EV-HLSCs stained for CD45 to identify the presence of inflammatory cells. Infiltrates of inflammatory CD45-positive cells (green) were present only in NASH mice that did not receive EV administration. Nuclei were counterstained with DAPI (blue). Original magnification, 400×. (D) Gene expression levels of anti-inflammatory cytokine *IL-10* in livers of healthy mice (CTRL) and of NASH mice treated with dose 2 of EV-HLSCs (EV-HLSCs) or with vehicle alone (NASH). Data are expressed as relative quantification using the ΔΔCt method. Normalization was made using GAPDH as a housekeeping gene. An ANOVA with Newman-Keuls multicomparison test was performed. **$p < 0.01$, NASH mice treated with vesicles (EV-HLSCs) versus NASH mice injected with vehicle alone (NASH).

patients with NAFLD,[33] reverted in mice treated with EVs, along with the expression levels of *Col1α1*, *Tgf-β1*, *Ltbp-1*, and *α-Sma*. In particular, *Ltbp-1*, which encodes for the protein responsible for activating *Tgf-β1* from its latent form to the active form and which is considered fundamental for fibrosis progression, was downregulated by EV treatment.[34]

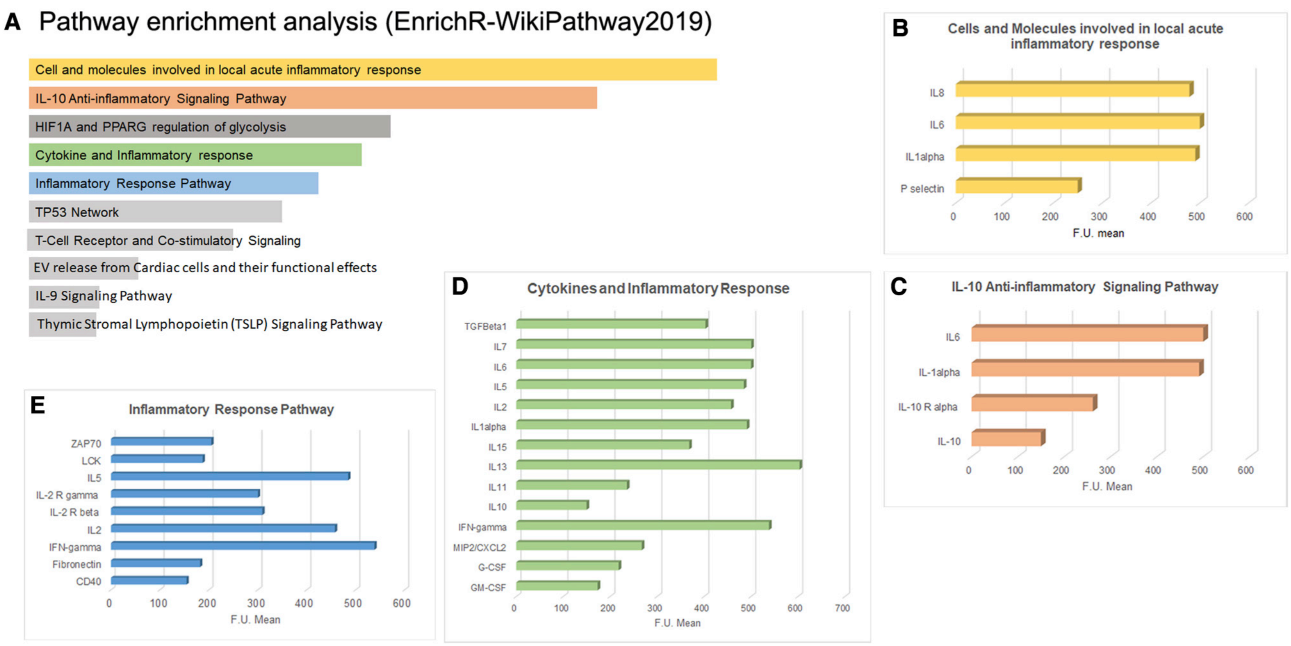

Figure 6. Pathway Enrichment Analysis of Proteins Vehicled by EV-HLSCs

(A) Representative bar graph showing the main pathways in which the 251 proteins vehicled by EV-HLSCs, identified by an antibody-based protein array, are involved. Most of them are related to inflammation and cytokine pathways. Data are ranked according to *Z* score and are reported in Table S2. (B–E) Bar graph showing the fluorescence intensity mean (F.U. mean) of the different proteins clustered in the main enriched pathways involved in inflammation: (B) proteins of local acute inflammatory response; (C) IL-10 anti-inflammatory signaling pathway; (D) cytokines and inflammatory response; and (E) inflammatory response pathway. The complete list of proteins with FU mean quantitation and pathway enrichment analysis are available in Table S3.

A key factor in the progression of NASH is the inflammation.[16–19] An infiltration of CD45-positive cells was observed in mice maintained with an MCDD for 4 weeks. NASH mice treated with EV-HLSCs had a significant reduction of CD45 cell accumulation within the liver, indicating the anti-inflammatory and immunomodulatory effects of EVs as detected at the molecular level. Moreover, the expression of IL-10, an anti-inflammatory and immunomodulatory cytokine,[35] was significantly enhanced after EV treatment.

We previously showed in two different models of chronic kidney diseases that the anti-fibrotic effect of EV-HLSCs is linked to the presence of microRNA (miRNA) patterns targeting the fibrotic genes.[10,11] In particular, we found that EV-HLSCs contain miRNA-29a, the let-7 family, miRNA-30a, miRNA-24, and miRNA-21, which are known to target Collagen I, Snail, and the FAS ligand.[11]

In the present study, we performed proteomic analyses of EV-HLSCs showing the presence of several anti-inflammatory proteins that may contribute to the final anti-fibrotic effect by downregulating inflammation. Therefore, EV-associated proteins may implement the biological activity of regulatory RNAs present in the EVs.[10,11]

In conclusion, the results of the present study indicated that EVs released from HLSCs exert anti-inflammatory and anti-fibrotic effects in a model of chronic liver disease by carrying molecules that may modulate genes involved in fibrosis. Fibrosis is an abnormal mechanism of tissue repair after repeated and sustained injuries that has common pathways in different organs. Therefore, taking into account the properties of EV-HLSCs, we can speculate that this may represent a potential therapeutic approach aimed to modify the abnormal response of different tissues to injury.

MATERIALS AND METHODS

HLSC Culture

HLSCs were obtained and cultured as previously described.[3,4] Briefly, HLSCs were generated by Anemocyte International (Gerenzano, Italy) from a 10- to15-mm liver fragment obtained from a liver donor, according to the standard criteria of Centro Nazionale Trapianti, as described previously.[3,4] The liver fragment was enzymatically digested with good manufacturing practice (GMP)-grade collagenase (NB 1, 0.6 mg/mL) and neutral protease (NBI, 0.73 mg/mL) for 30 min at 37°C. The liver cell suspension was washed (400 × g for 10 min) and cultured (2.5 × 10^5/mL in a T75 flask with 10 mL per flask) in the presence of minimal essential medium (α-MEM; Lonza, Basel, Switzerland) supplemented with 10% fetal calf serum (GIBCO, Cambrex), 10 ng/mL human recombinant epidermal growth factor (Miltenyi, Bergisch Gladbach, Germany), 10 ng/mL human recombinant basic fibroblast growth factor (Miltenyi, Bergisch Gladbach, Germany), 2 nM L-glutamine (Lonza), and 100 U/mL penicillin/streptomycin (Sigma, St. Louis, MO, USA) and maintained in a

humidified 5% CO_2 incubator at 37°C. After 2 weeks of culture, cells were seeded at a density of 2.5×10^5 cells per flask (T75) in the same culture medium for expansion.

EV-HLSCs: Purification and Characterization

EVs were obtained from supernatants of sub-confluent HLSCs cultured in serum-free α-MEM (EuroClone, Pero, Italy) for 18 h. Viability of cells at the time of supernatant collection was 98%, as confirmed by trypan blue exclusion. After the removal of cell debris and apoptotic bodies by centrifugation at 3,000 × *g* for 20 min and by microfiltration with 0.22-μm filters, EVs were purified by ultracentrifugation at 100,000 × *g* for 2 h at 4°C (Optima L-90 K; Beckman Coulter, Fullerton, CA, USA). The pellet of EVs obtained was resuspended in RPMI supplemented with 1% DMSO and stored at −80°C until use for subsequent studies.

For bio-distribution experiments, EV-HLSCs were labeled during ultracentrifugation with DiD fluorescent dye (Thermo Fisher Scientific, Waltham, MA, USA) as previously described.[36] One micromolar of Vybrant Cell Tracers DiD was added during the ultracentrifugation procedure. Then, labeled EVs were washed twice by ultracentrifugation in PBS (Lonza).

EV-HLSC number was determined using the NanoSight NS300 system (NanoSight, Amesbury, UK). EV-HLSC preparations were diluted (1:200) in sterile saline solution and analyzed using the NanoParticle Tracking Analysis (NTA) System with the NTA 3.2 Analytical Software as described previously.[36]

EV-HLSCs were characterized by cytofluorimetric analysis. Different EV-HLSC preparations (n = 3) were subjected to bead-based multiplex analysis by flow cytometry (MACSPlex Exosome Kit, human, Miltenyi Biotec).[14,15] Approximately 1×10^9 EV-HLSCs, quantified by NTA, were diluted with MACSPlex buffer (MPB) to a final volume of 120 μL and loaded into a 1.5-mL tube. After this, 15 μL MACSPlex Exosome Capture Beads (containing 39 different antibody-coated bead subsets) was added to each tube. For counterstaining of EVs bound by capture beads with detection antibodies, 5 μL each of APC-conjugated anti-CD9, anti-CD63, and anti-CD81 detection antibodies was added to each tube and then incubated in an orbital shaker for 1 h at 450 rpm at room temperature, protected from light. In this study, we mostly used a mixture of all three antibodies (pan-tetraspanin) in order to cover most EVs being present in the samples. In selected experiments, we used 5 μL APC-conjugated anti-CD29 (Miltenyi Biotec) antibody instead of the anti-tetraspanin antibodies. To wash the beads, 1 mL MPB was added to each tube and washed at 3,000 × g for 5 min. This was followed by another washing step with 1 mL MPB; incubation in an orbital shaker at 450 rpm, protected from light for 15 min at room temperature; and then washing at 3,000 × g for 5 min. After washing, 1 mL of the supernatant was carefully aspirated, leaving about 150 μL in the tubes, ready to be acquired.

Flow-cytometric analysis was performed with a CytoFLEX flow cytometer (Beckman Coulter, Brea, CA, USA). Approximately 5,000–8,000 single-bead events have been recorded per sample. Median fluorescence intensity (MFI) for all 39 capture bead subsets were background corrected by subtracting respective MFI values from matched media controls that were treated exactly like EV-containing samples (buffer/medium + capture beads + antibodies). All bead populations can be identified and gated based on their respective fluorescence intensity according to the manufacturer's instructions.

Transmission electron microscopy was performed on EVs placed on 200-mesh nickel formvar carbon-coated grids (Electron Microscopy Science, Hatfield, PA, USA) and left to adhere for 20 min, as previously described.[37] The grids were then incubated with 2.5% glutaraldehyde containing 2% sucrose and, after washings in distilled water, the EVs were negatively stained with NanoVan (Nanoprobes, Yaphank, NY, USA) and observed using a Jeol JEM 1010 electron microscope (Jeol, Tokyo, Japan).

Western Blot Analysis

Protein samples at a concentration of 10–30 μg were separated in 8% or 4%–15% gradient SDS-PAGE gels under reducing conditions and electroblotted onto 0.2-mm nitrocellulose membranes (GE Healthcare Life Sciences, Marlborough, MA, USA). The membranes were blocked in Tris-buffered saline-Tween 20 (TBS-T; 25 mM Tris [pH 8.0], 150 mM NaCl, and 0.05% Tween-20) containing 5% (w/v) non-fat dried milk for 1 h. After blocking, membranes were probed overnight with mouse anti-CD63, mouse anti-Alix (both from Santa Cruz Biotechnology, Santa Cruz, CA, USA), rabbit anti-CD9 (Abcam), and mouse anti-CD81 (Becton Dickinson). After extensive washings with TBS-T, the blots were incubated with the appropriate peroxidase-conjugated secondary antibodies for 1 h at room temperature. Following incubation, the membranes were washed extensively with TBS-T, probed with enhanced SuperSignal West Femto Maximum Sensitivity Substrate (Thermo Fisher Scientific), and detected with the ChemiDoc system (Bio-Rad, Hercules, CA, USA).

In Vivo Murine Model

Animal studies were conducted in accordance with the NIH Guide for the Care and Use of Laboratory Animals. All procedures were approved by the Italian Health Ministry (authorization number: 419/2016-PR).

To evaluate the ability of EV-HLSCs to improve liver fibrosis and inflammation, we induced NASH by continuously feeding the mice with an MCDD, as previously reported.[4,16–19] Male SCID mice (Charles River Laboratories, Wilmington, MA, USA), 10 weeks old, became accustomed to an MCDD (MP Biomedicals, Eschwege, Germany) through a mixture of standard and MCDD chow for 1 week. Thereafter, the full MCDD was given. Different amounts of EV-HLSCs were administered via i.v. injection (tail vein) twice a week, starting at week 2 of the diet (Figure 2), when fibrosis and inflammation have been established in the liver. Each mouse received a total of four EV injections. Different doses of EVs were tested: dose 1 (n = 9 mice), 5×10^9 EVs per mouse per injection; dose 2 (n = 9 mice), 2.5×10^9 EVs per mouse per injection; and dose 3 (n = 8

Table 1. Real Time PCR Specific Primers and Characteristics of Amplicons

Gene	Sense (5′ → 3′)	Antisense (5′ → 3′)
m-Col1α1	ACCTTGTTTGCCAGGTTCAC	ATCTCCCTGGTGCTGATGGAC
m-Tgf-β1	GCAACAATTCCTGGCGTTACC	CGAAAGCCCTGTATTCCGTCT
m-α-Sma	CATCTCCGAAGTCCAGCACA	GACGCACCACTGAACCCTAA
m-IL-1β	CAACCAACAAGTGATATTCTCCATG	GATCCACACTCTCCAGCTGCA
m-Ifn-γ	GAGCCAGATTATCTCTTTCATCC	GTTGTTGACCTCAAACTTGG
m-Tnf	CATCTTCTCAAAATTCGAGTGACAA	TGGGAGTAGACAAGGTACAACCC
m-IL-10	GACTTTAAGGGTTACTTGGGTTGC	TCCTGAGGGTCTTCAGCTTCTC
m-GADPH	TGTCAAGCTCATTTCCTGGTATGA	TACTCCTTGGAGGCCATGT

mice), 2.5×10^8 EVs per mouse per injection. The NASH control group was injected with vehicle alone (PBS; n = 23 mice). Control animals (n = 8) were fed with standard diet. All animals were sacrificed at week 4, and blood and liver were recovered for biochemical, histological, and molecular analyses.

In Vivo Bio-distribution of EV-HLSCs

Mice fed for 3 weeks with an MCDD and healthy mice were administered an i.v. injection of 3×10^{10} fluorescent EV-HLSCs, and their localization was monitored by OI (n = 6 per group). All the images have been acquired with the IVIS 200 small animal imaging system (PerkinElmer, Waltham, MA, USA) using an excitation filter at 640 nm and an emission filter at 700 nm.

Fluorescence analysis was performed by OI on dissected organs 3 h post-EV injection. Fluorescence emission was normalized to photons per second per centimeter squared per steradian (p/sec/cm^2/sr) as previously described.[36] The fluorescence signal in livers was quantified in region of interest (ROI) drawn freehand. Data were expressed as the average radiance ± SD. Images were acquired and analyzed using Living Image 4.0 software (PerkinElmer).

Histological Analyses

Liver morphology was evaluated through formalin-fixed paraffin-embedded tissue staining. Paraffin kidney sections (5 μm thick) were routinely stained for microscopic evaluation with H&E (Merck) or Sirius Red for collagen detection.

Liver fibrosis was quantified by measuring collagenous fibrotic areas stained in red (sections stained with Sirius Red) in 10 random fields per section from images taken at a magnification of 400×, using multiphase image analyses with ImageJ software v1.49.[38] The surface area occupied by steatosis vacuoles was quantified in 10 random fields per section from images taken at a magnification of 200×, using multiphase image analyses with ImageJ software. Immunofluorescence was performed on 5-μm-thick cryostat sections. Sections were stained with mouse anti-CD45 (Biorbyt, San Francisco, CA, USA) antibody for 2 h at 4°C. Rabbit anti-mouse Alexa Fluor 488 (Molecular Probes) was used as secondary antibody. Hoechst 33258 dye (Sigma) was added for nuclear staining. Confocal microscopy analysis was performed using a Zeiss LSM 5 Pascal model confocal microscope (Carl Zeiss International).

Molecular Analyses

Total RNA was extracted from liver tissue of control or NASH mice treated with or without EV-HLSCs using TRIzol Reagent (Ambion, Thermo Fisher Scientific, Waltham, MA, USA), according to the manufacturer's instructions. The TRIzol solutions were homogenized in a Bullet Blender instrument (Next Advance, New York, NY, USA) at a speed of 8 rpm for 3 min using 0.5-mm zirconium oxide beads and centrifuged at 12,000 × g for 15 min at 4°C. Supernatant from homogenized tissue was used to isolate RNA that was quantified spectrophotometrically (Nanodrop ND-2000, Thermo Fisher Scientific).

A total of 12 samples, four mice per experimental group, were retrotranscribed with the RT^2 First Strand Kit, and gene expression was analyzed using the Mouse Fibrosis RT^2 Profiler PCR Array (PAMM-120ZC; QIAGEN, Hilden, Germany) following the manufacturer's protocol. The analysis was performed with the online QIAGEN software using global normalization. Fold regulation expressions with respect to the control or NASH group were calculated for all samples using the ΔΔCt method.

Moreover, to confirm the expression of specific gene expression, quantitative real-time PCR was performed as described previously.[4] Briefly, 5 or 10 ng cDNA, retrotranscribed from RNA using the High Capacity cDNA Reverse Transcription Kit (Applied Biosystems, Foster City, CA, USA), were used in 20 μL reaction mixture containing sequence-specific oligonucleotide primers (purchased from MWG-Biotech, Eurofins Scientific, Brussels, Belgium) and the Power SYBR Green PCR Master Mix (Applied Biosystems) and were analyzed with StepOne Plus Real-Time PCR System (Applied Biosystems). GAPDH was used as the housekeeping gene to normalize RNA inputs. Fold change expressions with respect to control were calculated for all samples using the ΔΔCt method. The primers used for quantitative real-time PCR are reported in Table 1.

EV-HLSC Protein Content Analysis

EV-HLSC protein content was analyzed with the Human L1000 Array kit (RayBiotech, Peachtree Corners, GA, USA). EV-HLSCs

from two different pools were ultracentrifuged for 2 h at 100,000 × *g* at 4°C and then lysed with the lysis buffer provided by the kit following the manufacturer's instructions. Protein quantification was obtained with the Pierce BCA Protein Assay Kit (Thermo Fisher Scientific). Approximately 25 μg total protein was hybridized onto a glass slide according to the manufacturer's protocol. Fluorescence intensity was acquired, and background signal was subtracted for each point. Cutoff threshold was set at 150 fluorescent units (FUs). Data are expressed as FUs ± SD.

Pathway Enrichment Analysis

A list of protein vehicles by EV-HLSCs was subjected to bioinformatics analysis by EnrichR[39,40] online software. The software provided different databases, and WikiPathways 2019 was chosen because it was the most updated one. Pathways were listed and ranked after Fisher's exact test was performed for many random gene sets in order to compute a mean rank and SD from the expected rank for each term in the gene-set library and finally calculating a *Z* score to assess the deviation from the expected rank.

Statistical Analyses

Data analyses were performed using GraphPad Prism 6.0. Results are expressed as mean ± SD. Statistical analyses were performed by using an ANOVA with a Newman-Keuls test and Student's t test for bio-distribution experiments. A p value of < 0.05 was considered significant.

SUPPLEMENTAL INFORMATION

Supplemental Information can be found online at https://doi.org/10.1016/j.ymthe.2019.10.016.

AUTHOR CONTRIBUTIONS

S.B., M.B.H.S., and M.C. performed *in vivo* studies. S.B. performed cytofluorimetric characterization of EVs. C.P. and G. Chiabotto performed molecular tissue analysis. S.B. and M.B.H.S. performed histological analyses. M.T. and F.F. performed proteomic analyses of EVs. M.T. performed bioinformatics analyses. C.G. performed optical imaging experiments. M.C.D. performed western blot and electron microscopy analyses of the EV preparations. C.T. performed data interpretation and revised the manuscript. S.B. and G. Camussi performed study design, data interpretation, and manuscript writing. All authors approved the submitted the manuscript.

CONFLICTS OF INTEREST

C.T. is a full-time employee of Unicyte (Torino, Italy) and contributed to the study as a researcher. G. Camussi is a component of the scientific advisory board of Unicyte. M.B.H.S. and G. Camussi are named inventors in a related patent (WO2006126219-A1). The other authors declare no competing interests.

ACKNOWLEDGMENTS

This work was supported by a grant from Unicyte AG (Oberdorf, NW, Switzerland), a grant from "Terapie avanzate per processi fibrotici cronici" (Progetto EVER, codice domanda 320-40), and grant RiLo-2018. The authors thank Federica Antico for the technical assistance provided.

REFERENCES

1. Argo, C.K., and Caldwell, S.H. (2009). Epidemiology and natural history of non-alcoholic steatohepatitis. Clin. Liver Dis. *13*, 511–531.
2. Younossi, Z.M., Koenig, A.B., Abdelatif, D., Fazel, Y., Henry, L., and Wymer, M. (2016). Global epidemiology of nonalcoholic fatty liver disease—meta-analytic assessment of prevalence, incidence, and outcomes. Hepatology *64*, 73–84.
3. Herrera, M.B., Bruno, S., Buttiglieri, S., Tetta, C., Gatti, S., Deregibus, M.C., Bussolati, B., and Camussi, G. (2006). Isolation and characterization of a stem cell population from adult human liver. Stem Cells *24*, 2840–2850.
4. Bruno, S., Herrera Sanchez, M.B., Pasquino, C., Tapparo, M., Cedrino, M., Tetta, C., and Camussi, G. (2019). Human liver-derived stem cells improve fibrosis and inflammation associated with nonalcoholic steatohepatitis. Stem Cells Int. *2019*, 6351091.
5. Bruno, S., Grange, C., Tapparo, M., Pasquino, C., Romagnoli, R., Dametto, E., Amoroso, A., Tetta, C., and Camussi, G. (2016). Human liver stem cells suppress T-cell proliferation, NK activity, and dendritic cell differentiation. Stem Cells Int. *2016*, 8468549.
6. Fonsato, V., Herrera, M.B., Buttiglieri, S., Gatti, S., Camussi, G., and Tetta, C. (2010). Use of a rotary bioartificial liver in the differentiation of human liver stem cells. Tissue Eng. Part C Methods *16*, 123–132.
7. Navarro-Tableros, V., Gai, C., Gomez, Y., Giunti, S., Pasquino, C., Deregibus, M.C., Tapparo, M., Pitino, A., Tetta, C., Brizzi, M.F., et al. (2019). Islet-like structures generated in vitro from adult human liver stem cells revert hyperglycemia in diabetic SCID mice. Stem Cell Rev. Rep. *15*, 93–111.
8. Herrera, M.B., Fonsato, V., Bruno, S., Grange, C., Gilbo, N., Romagnoli, R., Tetta, C., and Camussi, G. (2013). Human liver stem cells improve liver injury in a model of fulminant liver failure. Hepatology *57*, 311–319.
9. Herrera Sanchez, M.B., Bruno, S., Grange, C., Tapparo, M., Cantaluppi, V., Tetta, C., and Camussi, G. (2014). Human liver stem cells and derived extracellular vesicles improve recovery in a murine model of acute kidney injury. Stem Cell Res. Ther. *5*, 124.
10. Kholia, S., Herrera Sanchez, M.B., Cedrino, M., Papadimitriou, E., Tapparo, M., Deregibus, M.C., Brizzi, M.F., Tetta, C., and Camussi, G. (2018). Human liver stem cell-derived extracellular vesicles prevent aristolochic acid-induced kidney fibrosis. Front. Immunol. *9*, 1639.
11. Grange, C., Tritta, S., Tapparo, M., Cedrino, M., Tetta, C., Camussi, G., and Brizzi, M.F. (2019). Stem cell-derived extracellular vesicles inhibit and revert fibrosis progression in a mouse model of diabetic nephropathy. Sci. Rep. *9*, 4468.
12. Nawaz, M., Fatima, F., Vallabhaneni, K.C., Penfornis, P., Valadi, H., Ekström, K., Kholia, S., Whitt, J.D., Fernandes, J.D., Pochampally, R., et al. (2016). Extracellular vesicles: evolving factors in stem cell biology. Stem Cells Int. *2016*, 1073140.
13. Derkus, B., Emregul, K.C., and Emregul, E. (2017). A new approach in stem cell research—exosomes: their mechanism of action via cellular pathways. Cell Biol. Int. *41*, 466–475.
14. Koliha, N., Wiencek, Y., Heider, U., Jüngst, C., Kladt, N., Krauthäuser, S., Johnston, I.C., Bosio, A., Schauss, A., and Wild, S. (2016). A novel multiplex bead-based platform highlights the diversity of extracellular vesicles. J. Extracell. Vesicles *5*, 29975.
15. Wiklander, O.P.B., Bostancioglu, R.B., Welsh, J.A., Zickler, A.M., Murke, F., Corso, G., Felldin, U., Hagey, D.W., Evertsson, B., Liang, X.M., et al. (2018). Systematic methodological evaluation of a multiplex bead-based flow cytometry assay for detection of extracellular vesicle surface signatures. Front. Immunol. *9*, 1326.
16. Pelz, S., Stock, P., Brückner, S., and Christ, B. (2012). A methionine-choline-deficient diet elicits NASH in the immunodeficient mouse featuring a model for hepatic cell transplantation. Exp. Cell Res. *318*, 276–287.
17. Winkler, S., Borkham-Kamphorst, E., Stock, P., Brückner, S., Dollinger, M., Weiskirchen, R., and Christ, B. (2014). Human mesenchymal stem cells towards non-alcoholic steatohepatitis in an immunodeficient mouse model. Exp. Cell Res. *326*, 230–239.
18. Chien, Y., Huang, C.S., Lin, H.C., Lu, K.H., Tsai, P.H., Lai, Y.H., Chen, K.H., Lee, S.D., Huang, Y.H., and Wang, C.Y. (2018). Improvement of non-alcoholic steatohepatitis

by hepatocyte-like cells generated from iPSCs with Oct4/Sox2/Klf4/Parp1. Oncotarget *9*, 18594–18606.

19. Machado, M.V., Michelotti, G.A., Xie, G., Almeida Pereira, T., Boursier, J., Bohnic, B., Guy, C.D., and Diehl, A.M. (2015). Mouse models of diet-induced nonalcoholic steatohepatitis reproduce the heterogeneity of the human disease. PLoS ONE *10*, e0127991.
20. Chen, L., Xiang, B., Wang, X., and Xiang, C. (2017). Exosomes derived from human menstrual blood-derived stem cells alleviate fulminant hepatic failure. Stem Cell Res. Ther. *8*, 9.
21. Haga, H., Yan, I.K., Takahashi, K., Matsuda, A., and Patel, T. (2017). Extracellular vesicles from bone marrow-derived mesenchymal stem cells improve survival from lethal hepatic failure in mice. Stem Cells Transl. Med. *6*, 1262–1272.
22. Haga, H., Yan, I.K., Borrelli, D.A., Matsuda, A., Parasramka, M., Shukla, N., Lee, D.D., and Patel, T. (2017). Extracellular vesicles from bone marrow-derived mesenchymal stem cells protect against murine hepatic ischemia/reperfusion injury. Liver Transpl. *23*, 791–803.
23. Nong, K., Wang, W., Niu, X., Hu, B., Ma, C., Bai, Y., Wu, B., Wang, Y., and Ai, K. (2016). Hepatoprotective effect of exosomes from human-induced pluripotent stem cell-derived mesenchymal stromal cells against hepatic ischemia-reperfusion injury in rats. Cytotherapy *18*, 1548–1559.
24. Yan, Y., Jiang, W., Tan, Y., Zou, S., Zhang, H., Mao, F., Gong, A., Qian, H., and Xu, W. (2017). hucMSC exosome-derived GPX1 is required for the recovery of hepatic oxidant injury. Mol. Ther. *25*, 465–479.
25. Tan, C.Y., Lai, R.C., Wong, W., Dan, Y.Y., Lim, S.K., and Ho, H.K. (2014). Mesenchymal stem cell-derived exosomes promote hepatic regeneration in drug-induced liver injury models. Stem Cell Res. Ther. *5*, 76.
26. Damania, A., Jaiman, D., Teotia, A.K., and Kumar, A. (2018). Mesenchymal stromal cell-derived exosome-rich fractionated secretome confers a hepatoprotective effect in liver injury. Stem Cell Res. Ther. *9*, 31.
27. Li, T., Yan, Y., Wang, B., Qian, H., Zhang, X., Shen, L., Wang, M., Zhou, Y., Zhu, W., Li, W., and Xu, W. (2013). Exosomes derived from human umbilical cord mesenchymal stem cells alleviate liver fibrosis. Stem Cells Dev. *22*, 845–854.
28. Mardpour, S., Hassani, S.N., Mardpour, S., Sayahpour, F., Vosough, M., Ai, J., Aghdami, N., Hamidieh, A.A., and Baharvand, H. (2018). Extracellular vesicles derived from human embryonic stem cell-MSCs ameliorate cirrhosis in thioacetamide-induced chronic liver injury. J. Cell. Physiol. *233*, 9330–9344.
29. Ohara, M., Ohnishi, S., Hosono, H., Yamamoto, K., Yuyama, K., Nakamura, H., Fu, Q., Maehara, O., Suda, G., and Sakamoto, N. (2018). Extracellular vesicles from amnion-derived mesenchymal stem cells ameliorate hepatic inflammation and fibrosis in rats. Stem Cells Int. *2018*, 3212643.
30. Jiang, W., Tan, Y., Cai, M., Zhao, T., Mao, F., Zhang, X., Xu, W., Yan, Z., Qian, H., and Yan, Y. (2018). Human umbilical cord MSC-derived exosomes suppress the development of CCl_4-induced liver injury through antioxidant effect. Stem Cells Int. *2018*, 6079642.
31. Chen, L., Chen, R., Kemper, S., Cong, M., You, H., and Brigstock, D.R. (2018). Therapeutic effects of serum extracellular vesicles in liver fibrosis. J. Extracell. Vesicles *7*, 1461505.
32. Vader, P., Mol, E.A., Pasterkamp, G., and Schiffelers, R.M. (2016). Extracellular vesicles for drug delivery. Adv. Drug Deliv. Rev. *106* (Pt A), 148–156.
33. Mesarwi, O.A., Shin, M.K., Drager, L.F., Bevans-Fonti, S., Jun, J.C., Putcha, N., Torbenson, M.S., Pedrosa, R.P., Lorenzi-Filho, G., Steele, K.E., et al. (2015). Lysyl oxidase as a serum biomarker of liver fibrosis in patients with severe obesity and obstructive sleep apnea. Sleep (Basel) *38*, 1583–1591.
34. Schlötzer-Schrehardt, U., Zenkel, M., Küchle, M., Sakai, L.Y., and Naumann, G.O. (2001). Role of transforming growth factor-beta1 and its latent form binding protein in pseudoexfoliation syndrome. Exp. Eye Res. *73*, 765–780.
35. Mosser, D.M., and Zhang, X. (2008). Interleukin-10: new perspectives on an old cytokine. Immunol. Rev. *226*, 205–218.
36. Grange, C., Tapparo, M., Bruno, S., Chatterjee, D., Quesenberry, P.J., Tetta, C., and Camussi, G. (2014). Biodistribution of mesenchymal stem cell-derived extracellular vesicles in a model of acute kidney injury monitored by optical imaging. Int. J. Mol. Med. *33*, 1055–1063.
37. Deregibus, M.C., Figliolini, F., D'Antico, S., Manzini, P.M., Pasquino, C., De Lena, M., Tetta, C., Brizzi, M.F., and Camussi, G. (2016). Charge-based precipitation of extracellular vesicles. Int. J. Mol. Med. *38*, 1359–1366.
38. Schneider, C.A., Rasband, W.S., and Eliceiri, K.W. (2012). NIH Image to ImageJ: 25 years of image analysis. Nat. Methods *9*, 671–675.
39. Chen, E.Y., Tan, C.M., Kou, Y., Duan, Q., Wang, Z., Meirelles, G.V., Clark, N.R., and Ma'ayan, A. (2013). Enrichr: interactive and collaborative HTML5 gene list enrichment analysis tool. BMC Bioinformatics *14*, 128.
40. Kuleshov, M.V., Jones, M.R., Rouillard, A.D., Fernandez, N.F., Duan, Q., Wang, Z., Koplev, S., Jenkins, S.L., Jagodnik, K.M., Lachmann, A., et al. (2016). Enrichr: a comprehensive gene set enrichment analysis web server 2016 update. Nucleic Acids Res. *44* (W1), W90–W97.

YMTHE, Volume *28*

Supplemental Information

HLSC-Derived Extracellular Vesicles Attenuate Liver Fibrosis and Inflammation in a Murine Model of Non-alcoholic Steatohepatitis

Stefania Bruno, Chiara Pasquino, Maria Beatriz Herrera Sanchez, Marta Tapparo, Federico Figliolini, Cristina Grange, Giulia Chiabotto, Massimo Cedrino, Maria Chiara Deregibus, Ciro Tetta, and Giovanni Camussi

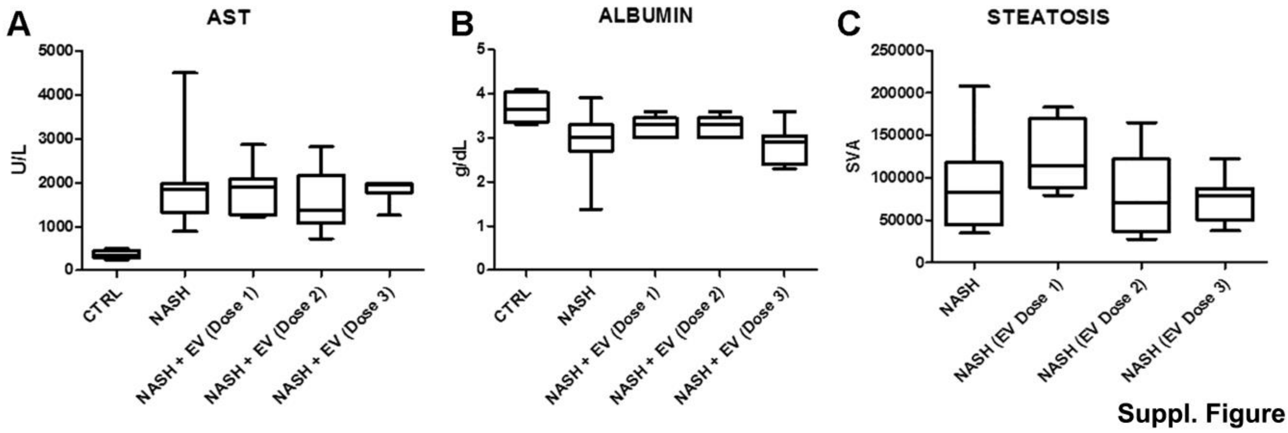

Suppl. Figure 1

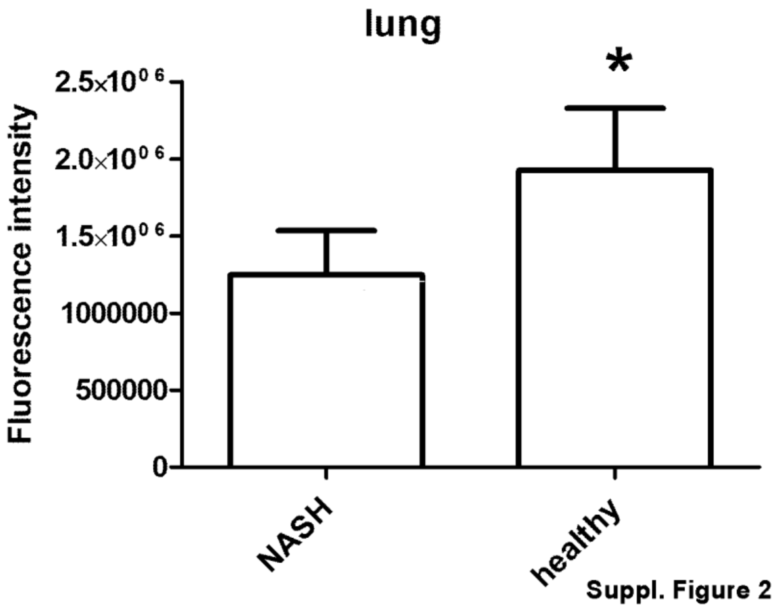

Suppl. Figure 2

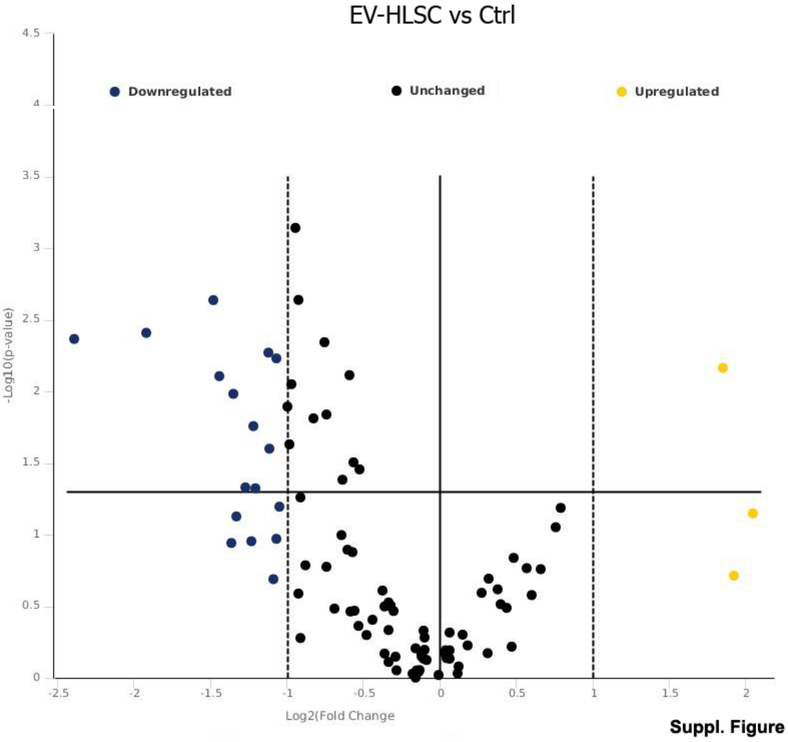

Suppl. Figure 3

Supplementary Figure 1: Effects of EV-HLSC administration on liver function and morphology in a murine model of NASH. Levels of aspartate aminotransferase (AST) (A), expressed as U/l, and albumin (B), expressed as g/dL, in serum of control mice (CTRL) and of mice fed with MCDD for 4 weeks treated with vehicle alone (NASH) or with iv injection of different doses of EVs (Dose 1: 5x109 EVs/mouse/injection, n=9; Dose 2: 2.5x109 EVs/mouse/injection, n=9 and Dose 3: 2.5x108 EVs/mouse/injection, n=8) and sacrificed at week 4. C) Histological quantification of the surface area occupied by steatosis vacuoles in MCDD-fed mice injected with different dose of EVHLSCs or with vehicle alone (NASH). Data shown for AST, Albumin and steatosis represents mean±SD. No statistical significant differences have been observed performing Anova with NewmanKeuls multicomparison test.

Supplementary Figure 2. Bio-distribution of EV-HLSCs. Quantification of fluorescence intensity in lungs of NASH and healthy mice injected with fluorescent EV-HLSCs. **p<0.01 healthy control mice vs NASH mice, student’s T Test.

Supplementary Figure 3. The volcano plots identify significant changes in gene expression between EV-HLSC-treated NASH (EV-HLSC) mice respect to control healthy group (Ctrl). The volcano plot displays the statistical significance (p<0.005) versus fold-change on the y- and x-axes, respectively.

Supplementary Table1. Fold Change values of genes analysed using Mouse Fibrosis PCR Array

	NASH vs Ctrl			EV-HLSC vs NASH			EV-HLSC vs Ctrl		
**Symbol**	**Fold Change**	**95% CI**	**p-value**	**Fold Change**	**95% CI**	**p-value**	**Fold Change**	**95% CI**	**p-value**
**Acta2**	2.3173	(0.82. 3.81)	0.009777	0.5354	(0.36. 0.71)	0.007	1.2407	(0.38. 2.10)	0.67304
**Agt**	0.778	(0.54. 1.02)	0.162262	1.1354	(0.47. 1.80)	0.54849	0.8834	(0.40. 1.37)	0.93658
**Akt1**	1.0354	(0.78. 1.29)	0.900035	0.6183	(0.40. 0.84)	0.03639	0.6402	(0.38. 0.90)	0.1006
**Bcl2**	1.1766	(0.71. 1.64)	0.447135	0.3911	(0.23. 0.55)	0.01927	0.4602	(0.32. 0.60)	0.00535
**Bmp7**	0.1734	(0.11. 0.24)	0.003927	1.1006	(0.80. 1.40)	0.52413	0.1908	(0.12. 0.26)	0.00429
**Cav1**	0.7828	(0.43. 1.13)	0.304835	0.941	(0.47. 1.42)	0.97574	0.7366	(0.32. 1.15)	0.39281
**Ccl11**	0.9932	(0.00001. 2.53)	0.951701	0.5349	(0.00001. 1.36)	0.52967	0.5312	(0.00001. 1.33)	0.52657
**Ccl12**	0.5895	(0.09. 1.08)	0.382763	0.8928	(0.00001. 2.00)	0.89343	0.5264	(0.01. 1.05)	0.25771
**Ccl3**	11.9747	(6.24. 17.71)	0.003656	0.3445	(0.10. 0.59)	0.04104	4.1258	(1.55. 6.70)	0.07098
**Ccr2**	7.6458	(5.83. 9.47)	0	0.2259	(0.13. 0.32)	1.1E-05	1.7269	(0.90. 2.55)	0.06502
**Cebpb**	0.5256	(0.31. 0.74)	0.041405	0.9202	(0.40. 1.44)	0.96215	0.4837	(0.20. 0.77)	0.06368
**Col1a2**	2.2113	(1.21. 3.21)	0.008617	0.2704	(0.11. 0.43)	0.00207	0.5979	(0.20. 0.99)	0.16768
**Col3a1**	1.9321	(1.03. 2.84)	0.021212	0.2469	(0.06. 0.43)	0.00173	0.4769	(0.08. 0.87)	0.10693
**Ctgf**	0.9463	(0.18. 1.72)	0.695945	0.7117	(0.06. 1.37)	0.42241	0.6735	(0.35. 0.99)	0.13247
**Cxcr4**	7.1934	(3.61. 10.78)	0.00001	0.5021	(0.34. 0.66)	0.00255	3.6117	(1.53. 5.69)	0.00683
**Dcn**	1.1346	(1.05. 1.22)	0.02177	0.9218	(0.79. 1.05)	0.30223	1.0458	(0.91. 1.18)	0.48205
**Edn1**	1.6794	(0.51. 2.85)	0.215387	0.5342	(0.25. 0.82)	0.06204	0.8971	(0.33. 1.46)	0.62207
**Egf**	0.6398	(0.41. 0.87)	0.0566	0.6722	(0.33. 1.01)	0.20214	0.4301	(0.20. 0.66)	0.01744
**Eng**	0.7227	(0.62. 0.82)	0.003473	0.5436	(0.24. 0.85)	0.112	0.3929	(0.18. 0.61)	0.01037
**Fasl**	5.3126	(1.94. 8.68)	0.030631	0.255	(0.09. 0.42)	0.04226	1.3549	(0.60. 2.11)	0.32428
**Grem1**	0.5758	(0.19. 0.97)	0.246986	1.3863	(0.52. 2.26)	0.57727	0.7982	(0.48. 1.11)	0.31432
**Hgf**	1.2463	(0.86. 1.63)	0.21641	0.6516	(0.37. 0.94)	0.11709	0.8121	(0.53. 1.10)	0.34099
**Ifng**	1.125	(0.08. 2.17)	0.431795	0.7303	(0.00001. 1.51)	0.47898	0.8215	(0.23. 1.41)	0.887
**Il10**	0.9133	(0.10. 1.72)	0.802507	1.19	(0.21. 2.17)	0.85417	1.0869	(0.72. 1.46)	0.83257
**Il13**	0.4836	(0.30. 0.67)	0.028099	1.6506	(1.19. 2.11)	0.02312	0.7982	(0.48. 1.11)	0.31432
**Il13ra2**	1.3238	(0.00001. 2.65)	0.95142	1.0477	(0.00001. 2.12)	0.55665	1.3869	(0.00001. 3.31)	0.60546
**Il1a**	1.1588	(0.73. 1.59)	0.416864	0.5541	(0.28. 0.83)	0.09484	0.6421	(0.42. 0.86)	0.04129
**Il1b**	1.9201	(0.96. 2.88)	0.098822	0.4654	(0.12. 0.81)	0.13233	0.8936	(0.38. 1.41)	0.99589
**Il4**	0.7353	(0.34. 1.13)	0.389147	1.5395	(0.35. 2.73)	0.35135	1.132	(0.30. 1.96)	0.59327
**Il5**	0.6931	(0.35. 1.04)	0.222117	1.1517	(0.66. 1.64)	0.63656	0.7982	(0.48. 1.11)	0.31432
**Ilk**	0.6871	(0.62. 0.75)	0.000159	0.9658	(0.73. 1.20)	0.89094	0.6636	(0.51. 0.81)	0.00768
**Inhbe**	1.2968	(1.10. 1.49)	0.011228	0.3934	(0.22. 0.56)	0.00046	0.5101	(0.28. 0.74)	0.00888
**Itga1**	0.9731	(0.78. 1.16)	0.831254	0.5598	(0.20. 0.91)	0.19797	0.5447	(0.20. 0.89)	0.16362
**Itga2**	1.4334	(0.37. 2.50)	0.348073	0.4647	(0.11. 0.82)	0.11441	0.666	(0.16. 1.17)	0.34357
**Itga3**	1.1982	(1.06. 1.34)	0.022281	0.3318	(0.08. 0.59)	0.02319	0.3975	(0.09. 0.70)	0.07446
**Itgav**	1.1242	(0.86. 1.38)	0.32439	0.4736	(0.21. 0.73)	0.03828	0.5324	(0.26. 0.81)	0.05489
**Itgb1**	0.867	(0.73. 1.01)	0.140229	0.6905	(0.46. 0.92)	0.07516	0.5987	(0.41. 0.79)	0.01447
**Itgb3**	1.5183	(0.92. 2.12)	0.064605	0.5133	(0.15. 0.88)	0.09911	0.7794	(0.31. 1.25)	0.67665
**Itgb5**	0.9778	(0.78. 1.17)	0.9253	0.6058	(0.42. 0.79)	0.02376	0.5924	(0.44. 0.74)	0.00452
**Itgb6**	0.5635	(0.20. 0.93)	0.188038	1.4054	(0.57. 2.24)	0.48223	0.7919	(0.48. 1.10)	0.29715
**Itgb8**	1.0003	(0.63. 1.37)	0.979587	1.0439	(0.34. 1.75)	0.65543	1.0442	(0.35. 1.74)	0.64171
**Jun**	1.512	(0.86. 2.16)	0.148051	0.5138	(0.40. 0.63)	0.00064	0.7768	(0.42. 1.13)	0.31744
**Lox**	6.2362	(4.07. 8.41)	0.000315	0.1649	(0.04. 0.28)	0.00072	1.0282	(0.28. 1.77)	0.64166

Ltbp1	2.3205	(1.45. 3.19)	0.009011	0.4774	(0.29. 0.67)	0.01459	1.1078	(0.80. 1.42)	0.49886
Mmp13	6.3838	(2.86. 9.90)	0.018047	0.189	(0.08. 0.29)	0.02081	1.2066	(0.84. 1.57)	0.25484
Mmp14	2.4819	(1.97. 2.99)	0.000535	0.3673	(0.14. 0.60)	0.015	0.9116	(0.36. 1.47)	0.88269
Mmp1a	6.9291	(4.95. 8.91)	0.000065	0.2276	(0.11. 0.35)	0.00042	1.577	(0.73. 2.43)	0.17395
Mmp2	2.2953	(1.30. 3.29)	0.004088	0.6459	(0.42. 0.88)	0.04072	1.4826	(0.73. 2.23)	0.17137
Mmp3	1.602	(0.34. 2.86)	0.314204	0.4466	(0.07. 0.82)	0.19749	0.7154	(0.20. 1.23)	0.50196
Mmp8	3.4484	(0.61. 6.29)	0.149496	0.3765	(0.04. 0.71)	0.19082	1.2983	(0.74. 1.86)	0.2407
Mmp9	0.9057	(0.00001. 2.22)	0.63655	0.519	(0.00001. 1.25)	0.35808	0.4701	(0.10. 0.84)	0.20458
Myc	1.3872	(0.00001. 3.21)	0.720767	0.4902	(0.27. 0.71)	0.02042	0.68	(0.00001. 1.59)	0.33985
Nfkb1	1.0404	(0.83. 1.25)	0.640613	0.4826	(0.27. 0.70)	0.01916	0.5021	(0.30. 0.71)	0.01274
Pdgfa	2.3865	(1.67. 3.10)	0.005366	0.5858	(0.36. 0.81)	0.05099	1.398	(0.92. 1.88)	0.14522
Pdgfb	2.7182	(1.43. 4.01)	0.025223	0.2914	(0.08. 0.50)	0.03021	0.792	(0.32. 1.26)	0.77502
Plat	6.9183	(0.90. 12.94)	0.003338	0.1569	(0.06. 0.26)	0.00275	1.0857	(0.00. 2.17)	0.93073
Plau	4.1265	(2.44. 5.81)	0.004185	0.2276	(0.04. 0.42)	0.00908	0.9391	(0.21. 1.67)	0.75017
Plg	0.3715	(0.33. 0.41)	0.000001	0.9641	(0.48. 1.45)	0.83407	0.3582	(0.18. 0.54)	0.0023
Serpina1a	0.2375	(0.15. 0.32)	0.000049	2.1905	(1.35. 3.03)	0.00608	0.5202	(0.41. 0.63)	0.00072
Serpine1	3.927	(1.61. 6.24)	0.019076	0.4304	(0.19. 0.68)	0.05025	1.6902	(0.86. 2.52)	0.08861
Serpinh1	1.3918	(1.01. 1.78)	0.06524	0.7352	(0.30. 1.17)	0.37712	1.0232	(0.45. 1.60)	0.66888
Smad2	0.8425	(0.63. 1.06)	0.232039	0.8229	(0.57. 1.08)	0.25449	0.6932	(0.51. 0.88)	0.03494
Smad3	0.7426	(0.57. 0.92)	0.041624	0.4947	(0.19. 0.80)	0.09233	0.3673	(0.15. 0.58)	0.00782
Smad4	0.7271	(0.56. 0.89)	0.023957	0.9296	(0.62. 1.24)	0.73963	0.6759	(0.49. 0.86)	0.03122
Smad6	1.1697	(0.90. 1.44)	0.246943	0.3315	(0.00001. 0.67)	0.04746	0.3877	(0.00001. 0.79)	0.11436
Smad7	0.8074	(0.52. 1.09)	0.308004	0.5265	(0.01. 1.04)	0.33705	0.4251	(0.03. 0.82)	0.11116
Snai1	1.675	(0.00001. 3.65)	0.85244	0.4124	(0.11. 0.71)	0.06901	0.6908	(0.00001. 1.48)	0.43384
Sp1	0.7739	(0.57. 0.98)	0.113732	0.5353	(0.18. 0.89)	0.2047	0.4143	(0.15. 0.68)	0.04669
Stat1	1.1085	(0.78. 1.44)	0.450519	0.5089	(0.31. 0.71)	0.0152	0.5642	(0.37. 0.76)	0.0154
Stat6	0.6947	(0.35. 1.04)	0.231383	0.3808	(0.19. 0.57)	0.02298	0.2645	(0.16. 0.37)	0.00389
Tgfb1	1.6919	(1.09. 2.30)	0.04588	0.311	(0.17. 0.45)	0.00823	0.5262	(0.37. 0.68)	0.00229
Tgfb2	1.1598	(0.53. 1.78)	0.457972	0.4105	(0.19. 0.63)	0.03432	0.4761	(0.34. 0.62)	0.00587
Tgfb3	2.1165	(1.21. 3.02)	0.002523	0.2045	(0.09. 0.32)	3.9E-05	0.4329	(0.13. 0.73)	0.04741
Tgfbr1	1.123	(0.82. 1.43)	0.533936	0.7069	(0.40. 1.02)	0.18515	0.7938	(0.41. 1.18)	0.46284
Tgfbr2	0.9967	(0.80. 1.20)	0.981178	0.5081	(0.28. 0.74)	0.02511	0.5064	(0.28. 0.73)	0.02334
Tgif1	1.6514	(1.25. 2.05)	0.012599	0.603	(0.43. 0.78)	0.01813	0.9958	(0.79. 1.20)	0.95521
Thbs1	3.5986	(1.72. 5.48)	0.008686	0.183	(0.09. 0.28)	0.00484	0.6585	(0.35. 0.96)	0.12748
Thbs2	2.7908	(1.44. 4.14)	0.001948	0.4711	(0.35. 0.59)	0.00223	1.3149	(0.69. 1.94)	0.30602
Timp1	12.3755	(0.00001. 27.25)	0.211976	0.3064	(0.00001. 0.78)	0.32557	3.7919	(0.00001. 7.76)	0.19331
Timp2	1.4423	(1.06. 1.82)	0.045588	0.6458	(0.46. 0.83)	0.03652	0.9315	(0.69. 1.17)	0.63683
Timp3	0.9086	(0.65. 1.17)	0.495181	0.6831	(0.25. 1.12)	0.4916	0.6207	(0.22. 1.02)	0.32832
Timp4	0.5591	(0.18. 0.93)	0.217489	1.382	(0.51. 2.26)	0.59346	0.7727	(0.48. 1.06)	0.24578
Tnf	6.2125	(3.48. 8.95)	0.002238	0.2443	(0.01. 0.48)	0.01523	1.5175	(0.07. 2.96)	0.2641
Vegfa	0.4725	(0.33. 0.62)	0.000808	0.9792	(0.40. 1.56)	0.86329	0.4627	(0.22. 0.70)	0.02507
Actb	1.3747	(1.27. 1.48)	0.00016	0.6744	(0.56. 0.79)	0.00165	0.9272	(0.76. 1.10)	0.46795
B2m	0.7518	(0.58. 0.93)	0.042485	1.6581	(0.97. 2.35)	0.042	1.2465	(0.77. 1.73)	0.20293
Gapdh	0.8799	(0.67. 1.09)	0.31751	1.1898	(0.95. 1.43)	0.15023	1.0469	(0.82. 1.28)	0.73474
Gusb	1.4665	(1.11. 1.83)	0.030327	0.6372	(0.47. 0.81)	0.02038	0.9344	(0.75. 1.12)	0.52235
Hsp90ab1	0.8108	(0.70. 0.93)	0.030274	1.0082	(0.52. 1.50)	0.73206	0.8174	(0.41. 1.23)	0.7112

Supplementary Table 2: List of protein vehicled by EV-HLSCs

Protein name	Gene name	F.U. Mean	F.U. SD
ApoC3	APOC3	66202,7	2198,6
GM-CSF R alpha	CSF2RA	2148,9	2746,6
LAG-3	LAG3	1986,4	270,6
Amylin	IAPP	753,9	65,3
BD-1	DEFB1	713,6	25,3
HGFR	MET	655,6	98,2
GDF11	GDF11	653,6	21,6
MSHa	MSX1	653,4	84,2
GDF9	GDF9	643,9	4,0
GDF3	GDF3	607,3	35,4
IL-21	IL21	605,4	92,7
IL-13	IL13	603,4	169,1
Granzyme A	GZMA	587,5	44,2
GDF5	GDF5	583,8	37,4
GDF8	MSTN	576,7	21,0
GRO	CXCL2	549,2	97,9
IFN-gamma	IFNG	538,3	9,3
IL-7	IL7	500,6	70,7
IL-6	IL6	500,2	77,0
IL-1alpha	IL1A	491,2	52,7
IL-5	IL5	484,6	9,6
IL-8	CXCL8	479,0	50,9
CNTF	CNTF	464,9	2,5
IL-2	IL2	457,6	64,5
TNF-alpha	LTA	454,6	200,4
CD71	TFRC	447,4	109,3
GDF1	GDF1	436,5	3,8
BAI-1	ADGRB1	436,2	383,1
ErbB4	ERBB4	428,5	0,5
CNTF R alpha	CNTFR	422,6	15,0
Complement component C2	C2	416,2	63,7
Mammaglobin A	SCGB2A2	405,4	47,5
Clusterin	CLU	404,1	109,6
TGF-beta 1	TGFB1	403,2	178,5
ANG-1	ANGPT1	402,4	232,9
ENPP2	ENPP2	388,6	95,4
CD30	TNFRSF8	386,5	49,4
TNF-beta	LTB	385,5	179,6

MIP-3 beta / CCL19	CCL19	385,2	59,9
GFR alpha-3	GFRA3	371,9	63,4
IL-15	IL15	368,4	67,7
EDG-1	S1PR1	367,8	17,6
BMP-7	BMP7	349,7	55,0
LH	LHCGR	346,2	121,5
CTACK	CCL27	339,2	18,3
Insulysin / IDE	IDE	334,7	420,2
CA19-9	MUC16	333,1	56,1
NPTX1	NPTX1	331,4	57,2
GREMLIN	GREM1	330,1	14,2
IL-23	IL23A	327,8	406,7
CCL28	CCL28	326,5	6,1
C-peptide	INS	325,6	117,6
Carboxypeptidase N subunit 2/CPN2	CPN2	324,8	98,3
ACK1	TNK2	324,6	0,6
VDUP-1	TXNIP	320,2	201,6
Troponin C	TNNC1	319,7	99,4
Cytokeratin 19	KRT19	319,0	117,3
HCR / CRAM-A/B	CCHCR1	318,1	110,4
Aldolase C	ALDOC	315,6	32,3
Lyn	LYN	314,4	102,5
Growth Hormone (GH)	GH1	311,2	16,1
Insulin R	INSR	310,8	53,1
IL-2 R beta	IL2RB	309,3	18,8
CD 163	CD163	308,9	143,5
CHI3L1	CHI3L1	308,8	157,5
FER	FER	308,7	137,3
Alpha Lactalbumin	LALBA	308,3	11,9
IL-9	IL9	306,3	24,5
TRA-1-60	PODXL	305,0	171,0
IL-1 R6 / IL-1 Rrp2	IL1RL2	301,8	76,7
Dkk-4	DKK4	301,7	15,7
FGF-16	FGF16	301,2	28,8

ALPP (alkaline phosphatase, placental)	ALPP	300,3	54,7
IL-2 R gamma	IL2RG	300,2	15,6
MIP-1beta / CCL4	CCL4	296,3	97,4
MCP-1 / CCL2	CCL2	293,0	95,0
Creatinine	CSH1	292,3	82,3
AGRP	AGRP	290,8	46,7
Ceruloplasmin	CP	289,4	107,7
TRA-1-81	PODXL	288,9	164,3
Aldolase A	ALDOA	287,1	40,5
Glut5	SLC2A5	285,0	32,1
APOA4	APOA4	282,6	32,8
MINA	RIOX2	281,0	42,1
Tec	TEC	279,7	117,7
GITR L / TNFSF18 L	TNFSF18L	278,8	9,5
Endoglin / CD105	ENG	278,2	0,9
BTC	BTC	277,6	34,6
FGF-10 / KGF-2	FGF10	276,2	28,9
SERPING1/Plasma protease C1 inhibitor	SERPING1	273,0	154,4
Btk	BTK	272,5	76,5
IL-1 RI	IL1R1	270,7	31,2
MIG / CXCL9	CXCL9	268,1	46,6
MMP20	MMP20	267,5	10,3
MIP 2 / CXCL2	CXCL2	267,3	43,3
Kallikrein 2	KLK2	266,4	124,1
CTLA-4 / CD152	CTLA4	264,0	13,1
Ubiquitin+1	UBB	262,9	147,8
IL-10 R alpha	IL10RA	261,9	40,7
Cryptic	CFC1	261,3	0,1
FGF-18	FGF18	261,1	44,8
FRK	FRK	260,8	34,2
BMP-5	BMP5	259,1	9,9

Angiopoietin-like 1	ANGPTL1	258,8	32,7
Alpha 1-Acid Glycoprotein/ORM	ORM1	257,7	26,1
PDGF R beta	PDGFRB	256,3	65,4
Follistatin	FST	253,4	24,4
GLP-1	ZGLP1	252,8	88,8
P-selectin	SELP	250,0	104,2
BNP	NPPB	248,7	27,0
Coagulation factor XIII A	F13A1	247,8	46,0
TXK	TXK	245,4	69,2
CBP /KAT3A	CREBBP	244,9	39,8
PARC / CCL18	CCL18	243,5	45,0
Angiostatin	PLG	243,5	20,5
EphA4	EPHA4	242,6	18,0
Ntn1	NTN1	242,1	111,4
Thrombospondin-2	THBS2	241,8	115,7
Chordin-Like 2	CHRDL2	240,3	19,6
IL-1 R4 /ST2	IL1RL1	239,6	9,9
Corticosteroid-binding globulin	SERPINA6	236,8	40,9
IL-11	IL11	235,7	10,8
Frizzled-1	FZD1	235,2	7,1
IL-17	IL17A	233,6	24,0
S100 A8/A9	S100A8	231,2	26,7
MMP-8	MMP8	231,0	89,6
Itk	ITK	230,7	33,5
MATK	MATK	229,8	76,5
PR Isoform B/NR3C3	PGR	229,6	65,8
SHBG	SHBG	228,5	135,7
LTF	LTF	228,5	13,5
IL-23p19	IL23A	228,1	111,3
IL-17RD	IL17RD	228,0	148,4
Galanin	GAL	226,6	95,8
BMP-8	BMP8B	223,5	11,3
L-selectin / CD62L	SELL	223,0	25,9
BNIP2	BNIP2	222,2	61,6
LRG1	LRG1	221,7	132,9
CV-2 / Crossveinless-2	BMPER	221,0	35,3
TRAIL R1	TNFRSF10A	220,2	88,1
FGF-11	FGF11	218,2	14,5

G-CSF	CSF3	218,1	24,0
Fyn	FYN	218,1	101,2
PEPSINOGEN I	PGC	217,9	66,2
Fibrinopeptide A	FGA	214,3	76,4
FGF-17	FGF17	214,0	13,0
Glut1	SLC2A1	213,9	31,5
GASP-2 / WFIKKN	WFIKKN1	211,8	2,4
Frizzled-3	FZD3	210,6	2,4
ACTH	POMC	210,3	20,2
SAA	SAA1	209,9	71,3
TYRO10	DDR2	209,7	115,3
TRAIL R2	TNFRSF10B	207,0	56,5
Sonic Hedgehog (Shh N-terminal)	SHH	205,2	43,1
ZAP70	ZAP70	204,9	45,6
GADD45A	GADD45A	204,3	96,5
NF1	NF1	202,9	89,6
SDF-1	CXCL12	201,7	15,9
Hepassocin	FGL1	200,2	1,4
LPS	IRF6	198,6	77,2
IL-13 R alpha 1	IL13RA1	197,6	24,0
CCL14 / HCC-1 / HCC-3	CCL14	197,6	15,3
IL-20 R alpha	IL20RA	197,2	21,0
Glut2	SLC2A2	197,1	12,4
MSP alpha-chain / MST1	MST1	196,4	70,8
SLPI	SLPI	195,4	97,7
IL-1 F10 / IL-1HY2	IL1F10	194,1	27,5
PI 3Kinase p85 beta	PIK3R1	193,8	61,3
MAC-1	ITGAM	193,3	41,7
TGF-beta 5	TGFB5	192,7	58,6
CA 125	MUC16	191,6	52,6
PDGF R alpha	PDGFRA	191,5	38,4
Calbindin	CALB1	191,5	46,9
AFP	AFP	191,4	41,9
PAI-1	SERPINE1	191,1	154,1
Netrin G2	NTNG2	190,7	61,2
FAM3B	FAM3B	190,7	12,8

hCG alpha	CGA	190,4	86,6
BAX	BAX	190,2	27,4
ADAMTS-17	ADAMTS17	190,1	4,1
GRP	GRP	189,1	98,1
Activin C	IHBC	188,9	76,3
LCK	LCK	187,3	8,7
HP/Haptoglobin	HP	186,2	78,6
FGF-13 1B	FGF13	186,2	13,0
IL-1 R8 / IL1RAPL1	IL1RAPL1	185,9	26,7
EV15L	EVI5L	185,3	70,3
CK-MB	CKB	185,3	3,9
PPARg2/NR1C3	PPARG	184,3	41,6
HSP27	HSPB1	183,6	28,0
FGF-5	FGF5	183,5	22,2
ASPH	ASPH	182,9	35,5
Fibronectin	FN1	181,9	105,3
FGF-20	FGF20	181,5	11,9
GASP-1 / WFIKKNRP	WFIKKN2	181,3	8,5
APC	APC	181,0	13,9
VGF/Neurosecretory protein VGF	VGF	180,5	83,8
MUSK	MUSK	179,3	66,6
Ferritin	FTL	178,3	87,6
FGFR1 alpha	FGFR1	178,0	70,4
CNDP1	CNDP1	177,9	36,0
XEDAR / TNFRSF27	EDA2R	177,4	84,6
Integrin alpha V	ITGAV	177,2	54,6
ABL1	ABL1	176,9	6,7
CD97	ADGRE5	176,6	7,6
EMAP-II / EML2	EML2	175,7	1,7
IL-17B R	IL17RB	175,2	46,9
Thyroid Peroxidase / TPX	TPO	173,7	84,8
BCAM	bcam	173,6	33,0
GM-CSF	CSF2	173,3	16,6
GMNN	GMNN	172,8	48,4
DR3 / TNFRSF25	TNFRSF25	171,2	4,2
PPP2R5C	PPP2R5C	170,7	62,3
CLC	CLC	169,9	19,8
LDL R	LDLR	167,9	59,7
p21	CDKN1A	167,2	47,5

TACI / TNFRSF13B	TNFRSF13B	166,8	69,7
Wilms Tumor 1	WT1	166,8	114,9
NRG3	NRG3	166,8	58,8
E-selectin	SELE	165,8	11,8
CRTH-2	PTGDR2	165,2	19,6
EphA3	EPHA3	164,8	30,2
TARC / CCL17	CCL17	164,2	84,4
FOXN3	FOXN3	164,0	38,7
Endostatin	COL18A1	163,8	7,7
ANGPTL3	ANGPTL3	163,7	67,8
TSH	TSHB	163,1	92,1
Calcitonin	CALCA	162,7	75,7
EphA6	EPHA6	160,6	20,0
Kremen-1	KREMEN1	160,5	26,9
Glut3	SLC2A3	159,7	2,6
Marapsin/Pancreasin	PRSS27	159,2	70,5
MMP-2	MMP2	158,6	56,5
hCGb	CGB3	158,3	78,3
EphA1	EPHA1	158,0	73,0
GSR (Glutathione Reductase)	GSR	157,8	65,1
FIH (Calcium Sensing Receptor)	CASR	157,4	66,5
ACE/CD143	ACE	156,9	54,6
Complement component C9	C9	155,4	39,9
Neurokinin-A	TAC1	154,4	58,8
CD40	CD40	154,3	6,6
CD59 complement fragment	CD59	153,6	38,0
HE4	WFDC2	152,2	64,1
ADAMTS-19	ADAMTS19	151,5	51,9
11b-HSD1	HSD11B1	151,4	29,3
FSH	FSHB	151,3	39,0
EphB3	EPHB3	150,9	90,3
Hepcidin	HAMP	150,3	12,4
Hck	HCK	150,1	1,7
OX40 Ligand / TNFSF4	TNFSF4	149,9	123,5
IL-10	IL10	149,8	7,4
